# Fast Inhibition Slows and Desynchronizes Mouse Auditory Efferent Neuron Activity

**DOI:** 10.1523/JNEUROSCI.0382-24.2024

**Published:** 2024-06-27

**Authors:** Matthew Fischl, Alia Pederson, Rebecca Voglewede, Hui Cheng, Jordan Drew, Lester Torres Cadenas, Catherine J. C. Weisz

**Affiliations:** ^1^Section on Neuronal Circuitry, National Institute on Deafness and Other Communication Disorders, NIH, Bethesda, Maryland 20892; ^2^NIDCD Data Science Core, National Institute on Deafness and Other Communication Disorders, NIH, Bethesda, Maryland 20892

**Keywords:** auditory, cochlear nucleus, efferent, globular bushy cell, inhibition, MNTB, olivocochlear, T-stellate cell

## Abstract

The encoding of acoustic stimuli requires precise neuron timing. Auditory neurons in the cochlear nucleus (CN) and brainstem are well suited for accurate analysis of fast acoustic signals, given their physiological specializations of fast membrane time constants, fast axonal conduction, and reliable synaptic transmission. The medial olivocochlear (MOC) neurons that provide efferent inhibition of the cochlea reside in the ventral brainstem and participate in these fast neural circuits. However, their modulation of cochlear function occurs over time scales of a slower nature. This suggests the presence of mechanisms that reduce MOC inhibition of cochlear function. To determine how monaural excitatory and inhibitory synaptic inputs integrate to affect the timing of MOC neuron activity, we developed a novel in vitro slice preparation (“wedge-slice”). The wedge-slice maintains the ascending auditory nerve root, the entire CN and projecting axons, while preserving the ability to perform visually guided patch-clamp electrophysiology recordings from genetically identified MOC neurons. The “in vivo-like” timing of the wedge-slice demonstrates that the inhibitory pathway accelerates relative to the excitatory pathway when the ascending circuit is intact, and the CN portion of the inhibitory circuit is precise enough to compensate for reduced precision in later synapses. When combined with machine learning PSC analysis and computational modeling, we demonstrate a larger suppression of MOC neuron activity when the inhibition occurs with in vivo-like timing. This delay of MOC activity may ensure that the MOC system is only engaged by sustained background sounds, preventing a maladaptive hypersuppression of cochlear activity.

## Significance Statement

Auditory brainstem neurons are specialized for speed and fidelity to encode rapid features of sound. Extremely fast inhibition contributes to precise brainstem sound encoding. This circuit also projects to medial olivocochlear (MOC) efferent neurons that suppress cochlear function to enhance detection of signals in background sound. Using a novel brain slice preparation with intact ascending circuitry, we show that inhibition of MOC neurons can also be extremely fast, with the speed of the circuit localized to the cochlear nucleus. In contrast with the enhancement of precision afforded by fast inhibition in other brainstem auditory circuits, inhibition to MOC neurons instead has a variable onset that delays and desynchronizes activity, thus reducing precision for a slow, sustained response to background sounds.

## Introduction

Encoding of acoustic stimuli is enhanced by active “cochlear amplification,” including electromotility of outer hair cells (OHCs; Dallos, 1992; Ashmore et al., 2010). Medial olivocochlear (MOC) neurons provide efferent feedback to inhibit OHCs (Fex, 1967; Mountain, 1980; Siegel and Kim, 1982; Guinan, 1996, 2010). The subsequent suppression of sound-evoked cochlear vibrations improves salient sound detection in noise, protects against noise-evoked damage, and may contribute to auditory attention (Winslow and Sachs, 1987; Kawase et al., 1993; Rajan, 1995; Reiter and Liberman, 1995; Guinan, 2011; Terreros et al., 2016). While cholinergic MOC synapses onto OHCs are well characterized, the synapses onto MOC neurons in the brainstem ventral nucleus of the trapezoid body (VNTB) are incompletely characterized. Recent work in positively identified MOC neurons in brain slices from transgenic mice has demonstrated that cochlear nucleus (CN) T-stellate cells provide ascending excitation, with descending excitation from the inferior colliculus (IC; Romero and Trussell, 2021). MOC neurons also receive afferent inhibition from the medial nucleus of the trapezoid body (MNTB) which delays spontaneous APs in vitro (Torres Cadenas et al., 2020) and may prevent MOC suppression of rapidly changing sounds (Torres Cadenas et al., 2022). MOC neurons exhibit high-frequency APs (>300 Hz) in vitro (Tong et al., 2013; Sinclair et al., 2017; Romero and Trussell, 2021), but in vivo, rarely fire APs faster than 100 Hz even with loud sound, and have variable AP latencies (Fex, 1962; Robertson, 1984; Robertson and Gummer, 1985; Liberman and Brown, 1986; Brown, 1989). This indicates that MOC neurons are not a simple reflex immediately activated by excitation from the CN. Rather, combined excitatory, inhibitory, and modulatory inputs may shape MOC activity.

Ascending auditory pathways have specializations for temporal precision and fidelity. In particular, the three-neuron pathway (AN→GBC→MNTB) that provides inhibition throughout the brainstem are among the fastest, most reliable neuronal circuits (Brand et al., 2002; Jercog et al., 2010; Roberts et al., 2013; Ford et al., 2015), although mice have fewer GBC specializations compared with other species (Stange-Marten et al., 2017). It is unknown whether GBC→MNTB projections to MOC neurons are also fast or how inhibitory timing affects MOC function.

Brain slices used for synaptic physiology studies allow extensive pharmacological manipulations but often sever long-distance, circuitous projections, losing the cellular interactions that govern the timing, strength, and plasticity of incoming neuron pathways. Therefore, we developed a novel asymmetric slice preparation, the “wedge-slice,” that maintains sound-evoked monaural ascending circuitry (Fischl and Weisz, 2020). This enables investigation into integration of ascending excitation and inhibition in MOC neurons. On the one side, the slice is thin to allow light penetration needed for visualization during whole-cell patch-clamp electrophysiology experiments from MOC neurons. On the other (contralateral) side, where primary excitatory and inhibitory inputs originate, the slice is thicker. This maintains the auditory nerve (AN) root into the CN, the full CN circuitry, and primary excitatory (direct) and inhibitory (via MNTB) axons from the CN to the VNTB where MOC neuron recordings are performed.

We compared synaptic inputs with MOC neurons stimulated at the midline (MdL) to bypass intrinsic CN circuitry versus stimulating the AN root to engage the full complement of CN circuits. We found that GBC→MNTB projections to MOC neurons are fast and temporally precise, similar to projections to other auditory nuclei. Both speed and precision can be attributed to GBC neurons compensating for reduced precision in later synapses and highlighting the “coincidence detector” function of GBCs (Rhode et al., 1983; Smith and Rhode, 1987; Joris et al., 1994). A computational MOC neuron model demonstrated that enhanced speed of the AN→GBC→MNTB→MOC inhibitory pathway measured during wedge-slice conditions has a larger effect on suppressing MOC activity compared with simple stimulation (MdL-stimulation). This increased inhibitory speed delayed APs in MOC neurons, while variability of inhibitory timing across the MOC neuron population results in stochastic activity that may provide smooth inhibition of cochlear function.

## Materials and Methods

### Ethical approval and animal housing

Animal procedures followed National Institutes of Health guidelines, as approved by the National Institute of Neurological Disorders and Stroke/National Institute on Deafness and Other Communication Disorders Animal Care and Use Committee. Preweaned mice postnatal age 13–21 (P13–P21) were used for experiments and were housed with parents and littermates before use. Mice were housed in the NIDCD animal facility with a 12 h light/dark cycle where food and water were provided *ad libitum*. Mice of both sexes were used for experiments. For consideration of sex as a biological factor, postsynaptic currents (PSCs) recorded during stimulation of the ventral acoustic stria (“midline stimulation”; MdL-stimulation) were analyzed and compared between the sexes (see below for complete methods). No significant differences were found for excitatory or inhibitory PSCs using the metrics of onset latency, onset jitter, rise time, decay tau, amplitude, and probability (Table 1). Datasets were therefore pooled.

**Table 1.** Values for metrics analyzed for PSCs evoked with midline (MdL) stimulation for comparison of sex as a biological factor

		Onset	Onset	Rise	Decay	Amplitude (nA)	Probability
		Latency (ms)	Jitter (ms)	Time (ms)	Tau (ms)		
Male	EPSCs (10)	2.11 ± 0.43	0.19 ± 0.08	0.61 ± 0.28	2.44 ± 0.50	−0.05 ± 0.02	0.48 ± 0.21
Female	EPSCs (11)	2.02 ± 0.67	0.41 ± 0.15	0.73 ± 0.20	2.23 ± 0.55	−0.04 ± 0.02	0.52 ± 0.31
Male	IPSCs (8)	5.01 ± 1.98	0.53 ± 0.08	1.26 ± 0.59	5.79 ± 2.85	−0.07 ± 0.02	0.39 ± 0.11
Female	IPSCs (10)	5.30 ± 1.26	0.44 ± 0.20	1.40 ± 0.32	3.34 ± 0.48	−0.05 ± 0.02	0.25 ± 0.16

*n* # for each group in parentheses represents # of peaks (clusters) analyzed. Data presented as median ± MAD. Comparisons made between sexes showed no significant differences with Mann–Whitney *U* test.

### ChAT-IRES-Cre x tdTomato mouse line

ChAT-IRES-Cre transgenic mice on either a C57BL/6J (RRID:IMSR_JAX:028861) or a C57BL/6N (RRID:IMSR_JAX:018957) background strain were crossed with tdTomato reporter mice (Ai14, Cre reporter allele inserted into Rosa 26 locus; RRID:IMSR_JAX:007914) to yield offspring heterozygous for each allele for experiments. These mice were used to target MOC neurons for patch-clamp recordings as previously described (Torres Cadenas et al., 2020).

### Atoh7/Math5 Cre x GCaMP6f mouse line

Atoh7/Math5 Cre mice (Yang et al., 2003; RRID:MGI:3717726) were crossed with GCaMP6f Ai95(RCL-GCaMP6f, RRID:IMSR_JAX:028865) mice for use in calcium imaging experiments of cochlear nucleus bushy cells.

### Brain slice preparation

Mice were anesthetized by carbon dioxide inhalation at a rate of 20–30% of chamber volume per minute and then decapitated. The brain was removed in cold artificial cerebrospinal fluid (aCSF) containing the following (in mM): 124 NaCl, 1.2 CaCl_2_, 1.3 MgSO_4_, 5 KCl, 26 NaHCO_3_, 1.25 KH_2_PO_4_, and 10 dextrose; 1 mM kynurenic acid was included during slice preparation. The recording solution was the same as the slicing solution, but excluding kynurenic acid. The pH was equal to 7.4 when bubbled with 95% O_2_/5% CO_2_. In experiments in which mini-postsynaptic potentials (mPSPs) were recorded, 1 µM tetrodotoxin (TTX) was included in the recording aCSF.

Asymmetric slices were obtained as previously described (Fischl and Weisz 2020). Briefly, the brainstem was carefully dissected from the skull to maintain a portion of the AN roots entering the cochlear nucleus, similar to methods used for thick slice preparations (Jercog et al., 2010; Roberts et al., 2013). Then, a wedge-shaped section was acquired using a stage with an adjustable angle such that the lateral edge of one hemisphere was ∼1–1.2 mm thick and contained the cochlear nucleus, and the opposite lateral edge was ∼200 μm, creating a thickness of ∼300–400 μm where MOC neurons were identified for patch-clamp experiments in the VNTB. An additional 300 μm slice was obtained rostral to the “wedge-slice” and used for additional experiments. For some experiments utilizing midline stimulation for evoked PSPs or for recordings of miniPSPs, symmetrical brain slices were prepared as previously described (Torres Cadenas et al., 2020). Sections were transferred to an incubation chamber and maintained at 35 ± 1°C for 30–60 min. Slices were then cooled to room temperature until used for experiments within 4 h of slicing. Wedge-slices were used immediately after a short recovery incubation (30 min) to improve cell viability which tends to diminish more rapidly than typical symmetrical slices due to reduced aCSF solution penetration in the larger tissue volume.

### Patch-clamp recordings

Sections were transferred to a recording chamber which was continuously perfused with aCSF at a rate of 5–10 ml/min. The bath temperature was held at 35 ± 1°C using an in-line heater (Warner) coupled to a temperature controller (Warner). The tissue was viewed using a Nikon Eclipse Ni-E microscope with an Apo LWD 25×/1.10 NA water-immersion objective attached to a Retiga Electro CCD camera (QImaging) operated using NIS Elements software (version 4.51.01). Epifluorescence illumination with red emission filters were used to locate MOC neurons in the VNTB for recordings. Targeted cells were then observed using DIC optics for patch-clamp recordings.

Pipettes for patch-clamp recordings were pulled from 1.5 mm borosilicate glass capillaries to resistances between 3 and 7 MΩ. For voltage-clamp experiments, an internal solution containing (in mM) 76 Cs-methanesulfonate, 56 CsCl, 1 MgCl_2_, 1 CaCl_2_, 10 HEPES, 10 EGTA, 0.3 Na-GTP, 2 Mg-ATP, 5 Na_2_-phosphocreatine, 5 QX-314, and 0.01 Alexa Fluor 488 hydrazide was used. The pH was adjusted to 7.2 with CsOH. This high internal [Cl^−^] solution was used to increase the driving force of inhibitory synaptic currents mediated by chloride ions at resting membrane potential to increase their amplitude and promote their detection. The reversal potential for Cl^−^ with this internal was −20 mV. For current-clamp experiments, an internal solution containing (in mM) 125 K-gluconate, 5 KCl, 1 MgCl_2_, 0.1 CaCl_2_, 10 HEPES, 1 EGTA, 0.3 Na-GTP, 2 Mg-ATP, 1 Na_2_-phosphocreatine, and 0.01 Alexa Fluor 488 hydrazide was used. The pH was adjusted 7.2 with 1 N KOH. Liquid junction potentials were empirically tested and were −6 mV for the CsCl internal solution and −9 mV for the KGlu solution. Membrane voltages presented in this paper are not corrected for these liquid junction potentials. Electrophysiology recordings were performed using a HEKA EPC10 amplifier controlled using PatchMaster NEXT (version 1.1). The recordings were sampled at 50 kHz and filtered on-line at 10 kHz. Series resistance was compensated between 60 and 85%. Cells with residual series resistance <5 MΩ were included for analysis. Residual series resistance was 2.70 ± 1.10 MΩ in cells where data were recorded (mean ± SD; *n* = 71 cells). Cells were voltage clamped at −60 mV unless stated otherwise. In current clamp, holding currents were injected to maintain the baseline membrane potential at −60 mV.

### Stimulation of AN and ventral acoustic stria

PSCs and postsynaptic potentials (PSPs) recorded in MOC neurons were evoked by electrical stimulation of axons using a bipolar tungsten electrode (World Precision Instruments). For AN-stimulation, the electrode was lowered onto the approximate center of the AN root diameter between the cut end of the nerve and its entry into the CN. For stimulation at the midline, the electrode was placed just lateral to the midline (contralateral hemisphere to the MOC neuron recording) near the ventral surface of the tissue onto fibers of the ventral acoustic stria. The stimulation was applied with an Iso-Flex Stimulus Isolation Unit (A.M.P.I.), and the intensity was adjusted to obtain consistent amplitude postsynaptic responses in MOC neurons (stimulation range 10–2,000 μA). In two experiments using AN-stimulation, the stimulus intensity was increased until the PSC latencies jumped to a shorter value (Figs. 3, 4), indicating direct recruitment of CN axons and bypassing of AN synapses onto CN cells. To isolate inhibitory currents in voltage clamp, the membrane potential was clamped at 0 mV, the approximate reversal potential for AMPA-mediated glutamatergic currents. Inhibitory inputs were blocked where indicated with bath application of strychnine (1 µM) and gabazine (SR95531, 50 µM).

### Miniature PSP recordings

For miniature PSP (mPSP) recordings, 1 µM tetrodotoxin (TTX, Alomone Labs) was included in the aCSF. Recordings were performed for several minutes to collect baseline (control) mPSPs. Inhibitory inputs were blocked using bath application of strychnine (1 µM) and gabazine (SR95531, 50 µM), and gap-free recordings were again taken during pharmacological manipulation. mPSPs were detected using MiniAnalysis software version 6.0.7 (Synaptosoft) using a threshold of 2× RMS noise. PSCs were then accepted or rejected based on the characteristic PSC waveform. The decay time constants of PSCs were calculated in MiniAnalysis software from individual events.

### Calcium imaging

Calcium imaging of activity in CN bushy cells was performed using the Atoh7/Math5 Cre mouse line crossed with a GCaMP6f mouse line (see above). Asymmetric wedge-slices were prepared as described above (experimental model details). aCSF used for recording calcium signals was modified to contain 2 mM CaCl_2_. Epifluorescence illumination with green emission filters were used to locate bushy cell neurons in the anteroventral cochlear nucleus (AVCN). The AVCN was targeted to increase the potential for imaging of globular bushy cells (GBCs) which project to the contralateral MNTB. Calcium signals were imaged using a Nikon Eclipse Ni-E microscope with an Apo LWD 25×/1.10 NA water-immersion objective in 2-photon excitation mode at 920 nm (Mai Tai HP, Spectra-Physics). A single focal plane was imaged for data collection. Imaging was performed at 3 Hz for 20 s using the resonant scanning galvos (Nikon Elements software version 4.51.01). Protocols consisted of 5 s of baseline data collection followed by AN-stimulation in three bouts (each bout is 20 pulses at 100 Hz), at 5 s intervals, followed by an additional 5 s after the third stimulation bout, and then at least 30 s without imaging or stimulation. Control data was acquired for several minutes before glutamate receptor blockers were applied. In a subset of experiments, CNQX (5 μM) was applied alone to block ionotropic glutamate receptors. In the remaining calcium imaging experiments, APV (50 μM) was also added to additionally block NMDA receptors. Acquisition protocols were repeated during blocker application. Blockers were applied for ∼10 min before washout. After washout, protocols were repeated to assess recovery of control conditions.

### Computational MOC neuron model

A model of a single MOC neuron was constructed using NEURON v8 (Carnevale and Hines, 2006) and Python 3. The neuron topology was generated from a published MOC neuron morphology [Brown and Levine (2008), their Fig. 9]. The number of segments was empirically determined to be 86, inserted using the nseg function, and compartments were organized into larger morphological groups including the soma (length, 33.6 µm; diameter, 6.1 µm), axon (length, 180.0 µm; diameter, 1 µm), and dendrites. The three primary dendrites projected from the soma (lateral: length, 12.8 µm; diameter, 1 µm), dorsal (length, 10.1 µm; diameter, 1 µm), and medial (length, 7.3 µm; diameter, 1 µm). Each of these three primary dendrites branched further into 8–46 dendrites with lengths from 1.5 to 31.4 µm and diameters from 1–2 µm. This detailed neuron topology allowed specific control of physiological properties. The uniform axial resistance was 210 Ohm-cm, and membrane capacitance was 1 µF/cm^2^. Channels were inserted into the membrane (Table 2) to recapitulate our experimental results. HCN channel reversal potential was set to −38 mV. The model was run at 35 C.

**Table 2.** Biophysical properties of a modeled MOC neuron compared with measured values recorded from MOC neurons

Channels	Location (segments)	Conductance
High threshold K^+^ (h.HT)	All	0.02 S/cm^2^
Low threshold K^+^ (h.LT)	All	12 mS/cm^2^
Low threshold K^+^ (h.kbl_LT)	All	0.6 ms^−1^
H-H type Na^+^ (h.na)	All	8 nS/cm^2^
HCN (h.Ih_400t8)	All	12 µS/cm^2^
High threshold K^+^ (h.HT)	Axon	0.32 mS/cm^2^
Low threshold K^+^ (h.LT)	Axon	6 cS/cm^2^
H-H type Na^+^ (h.na)	Axon	9 kS/cm^2^

PSPs were simulated at the soma to replicate recorded values. The model MOC neuron responded to synaptic inputs based on recorded mini-EPSPs (mEPSP) and mini-IPSPs (mIPSP) with output amplitudes and waveforms closely matching recorded values (mean ± SD; mEPSP: amplitude, 0.86 ± 0.23 mV; time constant of decay, 8.65 ± 1.43 ms; *n* = 6 neurons; mIPSP: amplitude, −1.57 ± 1.41 mV; time constant of decay, 18.1 ± 9.68 ms; *n* = 7 neurons; model MOC mEPSP: amplitude, 0.90 mV; time constant of decay, 8.21 ms; model MOC mIPSP: amplitude, −1.56 mV; time constant of decay, 14.4 ms).

Next, synaptic potentials were simulated within the model MOC neuron to mimic PSPs evoked from MdL-stimulation experiments both in control conditions and with pharmacological blockade of inhibitory synaptic inputs [MOC recording control evoked-PSP: amplitude, 1.88 ± 0.95 mV; time constant of decay, 10.7 ± 4.00 ms; *n* = 9 neurons, inhibition blocked evoked-PSP (EPSP only): amplitude, 2.20 ± 1.17 mV; time constant of decay, 14.1 ± 7.50 ms; *n* = 9 neurons; model MOC control evoked-PSP: amplitude, 1.64 mV; time constant of decay, 14.8 ms; model MOC inhibition blocked evoked-PSP (EPSP only): amplitude, 1.91 mV; time constant of decay, 16.4 ms]. To achieve these output parameters, model MOC neuron input values were as follows using the Exp2Syn mechanism: MOC model EPSP input parameters: rise time, 0.2 ms; time constant of decay, 6 ms; synaptic weight, 0.0015; reversal potential, 0 mV; resulting amplitude, 8.506 mV. The inhibitory PSP was designed so that simulation of EPSPs and IPSPs in the model replicated control MOC neuron current-clamp recordings of evoked PSPs. The model MOC IPSP output parameters were amplitude, −0.269 mV; time constant of decay, 14.8 ms. To achieve these values, the model MOC IPSP input parameters were as follows: rise time, 2.75 ms; time constant of decay, 3.64; synaptic weight, 0.00032; reversal potential, −90 mV; resulting amplitude, −0.536 mV.

PSPs with excitatory and inhibitory components were simulated with onset timing that systematically changed in 1 ms increments from excitation–inhibition latencies (E–I latency) from −10 (IPSPs precede EPSPs by 10 ms) to +10 (EPSPs precede IPSPs by 10 ms). E–I latencies were also simulated within the model to replicate recorded PSC latencies (Results). PSPs were then simulated within the model in trains of 20 pulses at 100 Hz, using either the excitation-only PSP or the combined PSPs with E–I latencies that systematically varied between −10 and +10, in 1 ms intervals.

### Experimental design and statistical analyses

#### Statistics for PSCs

Analysis of synaptic inputs to MOC neurons required classification of evoked PSCs as excitatory or inhibitory. The Clampfit software was used to detect individual PSCs for analysis. Latency to PSC onset, rise time, latency to PSC peak, amplitude, and decay time constant (tau decay) were measured for PSCs recorded at both –60 and 0 mV holding potential. A single exponential function fit was used to calculate the time constant of decay (τ). These data were then used for individual cell clustering analysis (below).

#### Individual cell clustering analysis

Clustering analysis was performed using PSC metrics in order to sort PSCs into statistically defined clusters. Clustering was performed with PSCs collected at both –60 and 0 mV holding potential, when available. The different holding potentials were used to distinguish between EPSCs and IPSCs. At −60 mV, both EPSCs (reversal potential ∼0 mV) and IPSCs (60 mM internal chloride concentration, reversal potential approximately −20 mV to enhance IPSC amplitude for detection of inhibitory synaptic events) were inward and could not be distinguished based on polarity alone. At 0 mV, EPSCs were not visible and IPSCs were outward, allowing classification of recorded PSCs as inhibitory. PSC clusters recorded at −60 mV but not at 0 mV were classified as excitatory. For each cell's PSCs, values for onset latency, rise time, amplitude, and decay time constant collected from Clampfit were imported into R. Function libraries utilized for clustering included {parameters}, {factoextra}, and {NbClust}. The appropriate number of clusters was determined using the gap statistic method (Tibshirani et al., 2001). This method was chosen because of its ability to select “one” as the optimal number of clusters where appropriate. The “clusGap” function was used with kmax = 10 (max# of clusters), nstart = 25 (# of random start centers), and B = 500 (bootstrapping). Once the appropriate number of clusters was determined, a *k*-means cluster analysis was performed in *R* using the “kmeans” function to sort the PSCs into clusters (with centers = output # from gap statistical analysis and nstart = 25). Once the PSCs were assigned a cluster number, statistical analyses were performed on each cluster. For PSCs collected with midline stimulation where data was acquired at 0 mV holding potential, a cluster was deemed inhibitory if it was present at both −60 and 0 mV. A cluster was categorized as excitatory if PSCs from the cluster were only present at −60 mV. This was a robust categorization, with only 2 out of 11 cells having a PSC misidentified in the cluster. In both cells, a single excitatory PSC was classified into an inhibitory cluster (2 out of 654 misidentified PSCs). PSCs acquired with AN-stimulation were analyzed via the same cluster analysis. AN-stimulation PSCs were categorized as excitatory or inhibitory using a machine learning algorithm (see below). Statistical comparisons between excitatory and inhibitory PSC clusters were then made across the population.

#### Machine learning algorithm to classify PSCs

A RandomForest machine learning algorithm was utilized to classify recorded PSCs as excitatory or inhibitory based on the variables of PSC rise time, time constant of decay, amplitude, probability of occurrence within a cluster, onset jitter within a cluster, peak jitter within a cluster, and animal age. All values were continuous except for animal age, which was treated as a categorical variable. The model included 952 PSCs recorded in the ML-stimulation configuration at a holding potential of −60 mV, from 22 MOC neurons. These PSCs had been previously classified as excitatory or inhibitory based on recordings from the same neurons at a holding potential of 0 mV and the clustering analysis described above (PSC clusters recorded at a holding potential of −60 mV but not 0 mV were defined as excitatory; PSC clusters recorded both at holding potentials of −60 and 0 mV were defined as inhibitory). After training the RandomForest algorithm on this data, the model classification accuracy reached 99.89%, with out-of-bag (OOB) error stabilization at 150 “trees.” Rerunning the algorithm on the training dataset determined that it was able to distinguish excitatory and inhibitory events perfectly with an area under the curve (AUC) of 1, indicating excellent ability of the model to distinguish excitatory versus inhibitory PSCs. The model was then used to classify the individual PSCs in the AN-stimulation dataset recorded at −60 mV as excitatory or inhibitory. First, the RandomForest algorithm determined the probability that each of the 344 PSCs was excitatory or inhibitory PSCs that were given the classification that had the highest probability (>0.5) by the algorithm. The algorithm gave slightly higher classification probabilities for excitatory (0.81 ± 0.14; *n* = 181) compared with inhibitory (0.75 ± 0.12; *n* = 163) PSCs. AN-stimulation PSCs were grouped into 28 clusters determined above through cluster analysis. Three clusters contained both excitatory and inhibitory PSCs. Two cells had a majority of one classification (7 of 10 excitatory and 19 of 21 excitatory and one cell was approximately split 6 of 11 excitatory). For further analyses, we separated these three mixed clusters into an excitatory and inhibitory cluster each to yield a total of 31 AN-stimulation clusters.

#### Calcium imaging analysis

After acquisition of fluorescent signals in cochlear nucleus bushy cells in Atoh7/Math5 Cre; GCaMP6f wedge-slices (see above), fluorescence changes in response to electrical stimulation of axons were measured to determine the effect of synaptic stimulation of bushy cells with and without blockers of postsynaptic receptors. Polygonal ROIs were drawn by hand around neurons and any major processes that could be resolved (Elements software version 4.51.01), and average intensity values for ROIs were calculated for each frame. Maximum fluorescence elicited from AN-stimulation within a protocol was compared with baseline average (*F*) of each ROI. Fluorescence change (Δ*F*) and relative fluorescence change (Δ*F* / *F*) was calculated using Excel. Heat maps were constructed from the intensity value output of a given frame from the Elements software using custom MATLAB scripts. Baseline values were calculated as the average pixel intensity of the first 15 frames (∼5 s) before axon stimulation. Cells were considered active if the average fluorescence of an ROI reached two standard deviations (SDs) above the baseline average in at least two of the three stimulations during a protocol. Active cells were then used to compare the ΔF/F between control and glutamate block conditions.

#### Data analysis and statistics

Statistical analyses were performed in Origin (v2021 and v2022). Normality tests were performed on datasets using the Shapiro–Wilk test. The majority of datasets were non-normally distributed, so nonparametric testing was employed. The Mann–Whitney *U* test was used for testing between two independent groups. A one-sample Wilcoxon signed-rank test determined whether E–I latency difference values were significantly different from zero for MdL- and AN-stimulation PSCs. Action potential metrics collected at different stimulus rates (Fig. 6) were compared in the control condition using Friedman's ANOVA. Post hoc Dunn's test was used to test significance between stimulus rates. Action potential metrics were also compared between control and inhibition block conditions using paired Wilcoxon signed-rank test. Calcium imaging data were analyzed using Kruskal–Wallis ANOVA where post hoc Dunn's test was used to test whether control datasets between the CNQX and CNQX + APV conditions were significantly different from each other. Population data are summarized in box plots with the box representing the first and third quartiles, the line representing the median, the square representing the mean, and the error bars representing the 10th and 90th percentiles. The figures were prepared in Origin and Adobe Illustrator.

## Results

### Midline stimulation in wedge-slice preparations evokes mixed excitatory and inhibitory PSCs

**Figure 1.**
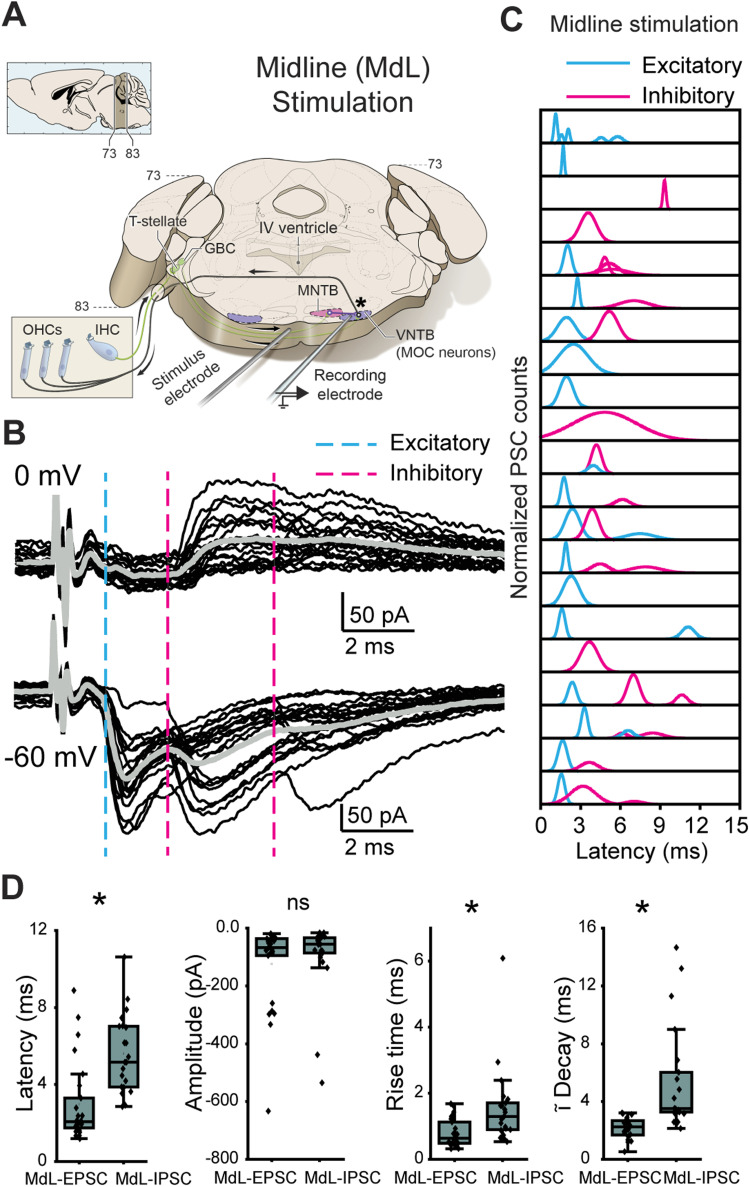
Stimulation of axons at the midline of the ventral brainstem evokes multicomponent PSCs in MOC neurons. ***A***, Schematic of wedge-slice preparation showing monaural circuitry of MOC neurons from ascending AN axons from the cochlea into the CN, excitatory (green) T-stellate and GBC projections to the SOC, inhibitory (purple) projections from the MNTB to the VNTB containing MOC neurons, and MOC axons (black) projecting back to cochlear OHCs. Note: the cochlea is not present in the recording preparation. Top left, Schematic indicates atlas coordinates of position along rostral-caudal extent of thick portion of the slice (Paxinos and Watson, 2001). Schematic by Alan Hoofring, NIH Medical Arts Branch. ***B***, Voltage-clamp traces from an identified MOC neuron during MdL-stimulation (1 Hz), evoking PSCs in multiple clusters at a holding potential of 0 mV (top) and −60 mV (bottom). Twenty sweeps overlaid (black), with gray line indicating the average waveform. Dashed lines indicate the approximate onset of PSCs in clusters of excitatory (blue) and inhibitory (magenta) PSCs. ***C***, Gaussian distributions fitted to frequency histograms (normalized) of the latency to PSC onset for clusters of MdL-EPSCs (blue) and MdL-IPSCs (magenta). Each row represents PSCs recorded in a different MOC neuron. ***D***, Comparison of parameters observed in MdL-EPSCs and MdL-IPSCs.

Ascending axons conveying acoustic information from the CN throughout the SOC traverse near the ventral surface of the brainstem. This includes axons projecting to MOC neurons in the VNTB that provide sound-evoked excitation, primarily T-stellate and possibly small cell cap (SCC) neurons (De Venecia et al., 2005; Darrow et al., 2012; Brown et al., 2013; Romero and Trussell, 2021; Hockley et al., 2022). This also includes axons of GBCs which provide sound-evoked inhibition via intervening MNTB neurons (Torres Cadenas et al., 2020). To investigate the convergence of these pathways, whole-cell patch-clamp recordings were performed in voltage clamp from positively identified MOC neurons in brainstem slices from P14 to P19 ChAT-IRES-Cre; tdTomato mice of both sexes. We used a combination of uniformly thick (300 µm) slices cut at ∼15° off the coronal plane (Torres Cadenas et al., 2020) and wedge-slices (Fischl and Weisz, 2020; Fig. 1*A*) while electrically stimulating close to the ventral surface of the slice near the midline (MdL) to evoke neurotransmitter release from presynaptic axons. These axons likely originated in the CN contralateral to the MOC neuron. With this technique, both excitatory and inhibitory circuits originating at the contralateral CN can be simultaneously stimulated. MdL-stimulation in this location is expected to directly activate T-stellate cell axons resulting in monosynaptically evoked, short latency excitatory postsynaptic currents (midline-evoked EPSCs: MdL-EPSCs). Additionally, stimulation of GBC axons accessed from the same location results in inhibitory postsynaptic currents (midline-evoked IPSCs: MdL-IPSCs) evoked via the disynaptic GBC→MNTB→MOC pathway that inhibits MOC neurons (Torres Cadenas et al., 2020). Other as-yet uncharacterized synaptic inputs to MOC neurons may also be activated with this technique. Consistent with activation of multiple classes of presynaptic axons, MdL-stimulation resulted in multicomponent PSCs in 13/21 recordings (Fig. 1*B*). PSCs occurred with a short latency from stimulation [1.85 ± 0.53 ms; *n* = 557 PSCs in 21 neurons; a subset of PSC latency data was previously published (Fischl and Weisz, 2020)].

With the high intracellular chloride concentration used in voltage-clamp recordings to maximize detection of IPSCs (intracellular chloride concentration, 60 mM; chloride reversal potential, −20 mV), both “excitatory” and “inhibitory” PSCs were inward at a membrane holding potential of −60 mV and therefore indistinguishable based on polarity. To distinguish between MdL-EPSCs and MdL-IPSCs, in all cells the MOC neuron holding potential was alternatively set to the AMPA receptor reversal potential of 0 mV to isolate outward, chloride-mediated PSCs that can be classified as “inhibitory” that are likely either GABAergic or glycinergic. In each experiment, stimulation was performed repeatedly (20–80 stimulations; Fig. 1*B*), and in each sweep, PSCs were detected and analyzed for parameters of onset latency, rise time (10–90% of peak), amplitude, and time constant of decay. These parameters (excluding amplitude) were used to sort PSCs into statistically defined clusters using *k*-means analyses (Materials and Methods). Clusters observed at both –60 and 0 mV holding potentials were classified as “inhibitory” MdL-IPSCs and clusters observed only at –60 mV were classified as “excitatory” MdL-EPSCs (Fig. 1*C*, each peak indicates a different “cluster”).

Following classification of PSCs as “excitatory” or “inhibitory” based on the presence or absence of the PSC cluster at a holding potential of 0 mV, we further characterized PSCs recorded at −60 mV by MdL-stimulation. Latency to the first EPSC was shorter than the latency to the first IPSC (MdL-EPSC latency: 1.92 ± 0.37, *n* = 18 cells; MdL-IPSC latency: 4.47 ± 0.93, *n* = 15 cells; Mann–Whitney *U* test; *p* = 4.79 × 10^−6^). MdL-EPSCs and MdL-IPSCs had similar amplitudes at −60 mV (MdL-EPSC: 66.95 ± 3.95 pA, *n* = 25 clusters, *n* = 17 cells; MdL-IPSC amplitude: 55.39 ± 3.10 pA, *n* = 21 clusters, *n* = 15 cells, Mann–Whitney *U* test; *p* = 0.69). MdL-EPSCs had faster kinetics than MdL-IPSCs, consistent with earlier work (Torres Cadenas et al., 2020; MdL-EPSC rise time: 0.63 ± 0.19 ms, *n* = 25 clusters, *n* = 17 cells; MdL-IPSC rise time: 0.86 ± 0.15 ms, *n* = 21 clusters, *n* = 15 cells). Mann–Whitney *U* test; *p* = 8.02 × 10^−4^; MdL-EPSC time constant of decay: 2.23 ± 0.52 ms, *n* = 23 clusters, *n* = 17 cells; MdL-IPSC time constant of decay: 3.51 ± 0.93 ms, *n* = 21 clusters, *n* = 15 cells Mann–Whitney *U* test; *p* = 4.95 × 10^−7^). Patterns of PSCs varied somewhat across MdL-stimulation experiments. Patterns included recordings with EPSCs only (6/21), IPSCs only (4/21), a single group of EPSCs, and a single group of IPSCs (5/21 recordings), and more complex patterns consisting of multiple EPSCs and/or multiple IPSCs (6/21; Fig. 1*C*). In all recordings with both EPSCs and IPSCs (11/21), EPSCs always occurred with a shorter latency than IPSCs. This shorter latency was expected given that the MdL-EPSC pathway is monosynaptic (T-stellate→MOC) while the MdL-IPSC pathway is disynaptic (GBC→MNTB→MOC), consistent with an extra synapse in the MdL-IPSC pathway incurring a delay.

### AN-stimulation–evoked PSCs

**Figure 2.**
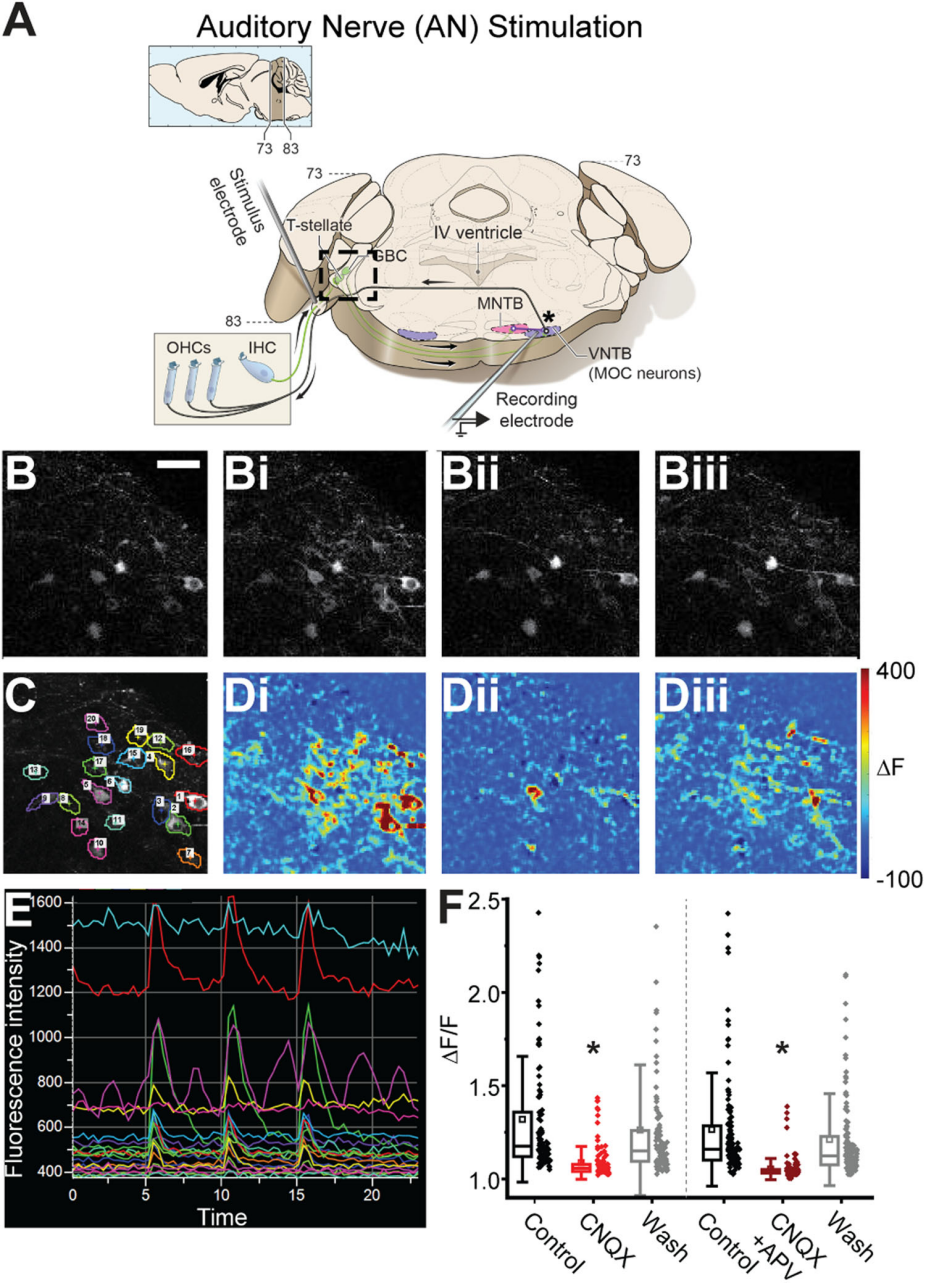
AN-stimulation in a wedge-slice evokes activity via synaptic activation of CN neurons. ***A***, Schematic of a wedge-slice, as in Figure 1, with stimulation at the AN root (AN-stimulation). Note: the cochlea is not present in the preparation. ***B***, Calcium imaging of bushy cells in Atoh7/Math5^Cre^; GCaMP6f mice during AN-stimulation in the following: ***B***, baseline (no stimulation); ***Bi***, control AN-stimulation at 100 Hz, 20 pulses; ***Bii***, AN-stimulation with blockers of AMPA and NMDA receptors (5 µM CNQX, 50 µM APV) in the aCSF; ***Biii***, AN-stimulation after wash of AMPA and NMDA receptor blockers. ***C***, Same CN imaging region as in ***B***, annotated with ROIs over individual bushy cells used for fluorescence analysis. ***D***, Heat maps showing change in fluorescence in same region as ***B*** and ***C*** during AN-stimulation; color scale indicates fluorescence change from baseline in the following: ***Di***, control; ***Dii***, AMPA and NMDA block; and ***Diii***, wash. ***E***, Raw fluorescence intensity changes for ROIs in ***C*** during three “bouts” (bout: 20 pulses, 100 Hz) of AN-stimulation in control conditions. ***F***, Quantification of fluorescence changes of bushy cells in response to AN-stimulation in control, glutamate receptor blockade (CNQX or CNQX + APV), and wash. Scale bar in ***B*** (50 μm) applies to all panels in ***B–D***.

The above experiments detail the relative timing of synaptic inputs to MOC neurons evoked simultaneously at the MdL from monosynaptic excitatory and disynaptic inhibitory inputs. The timing of synaptic inputs in the above experiments with MdL-stimulation is artificial because the full complexity of ascending circuitry is not activated in this stimulation paradigm, including AN synapses onto CN neurons, intrinsic CN circuits, and the potential differential propagation of APs down the specialized CN axons. To test the integration of ascending, monaural, excitatory, and inhibitory synaptic inputs to MOC neurons with a more in vivo-like timing, PSCs were evoked in MOC neurons by stimulating the contralateral AN root in wedge-slice preparations (Fig. 2*A*), which have an intact CN.

To ensure that electrical stimulation indeed excited CN neurons via synaptic activation, and not direct electrical activation which would bypass the strength and timing of AN→CN synapses, we performed two-photon calcium imaging of bushy cells in wedge-slices from Atoh7/Math5^Cre^ mice (Yang et al., 2003; Kronander et al., 2017) expressing GCaMP6f (Chen et al., 2013) in bushy cells, to measure suprathreshold activation during AN-stimulation. AN axons were stimulated in control conditions and with bath application of glutamate receptor antagonists to block synaptic transmission at AN→CN synapses. If the AN electrical activation stimulates glutamate release from AN axons to synaptically activate bushy cells, blockade of the glutamate receptors is expected to reduce the somatic calcium response. However, if AN electrical stimulation directly evokes APs in bushy cells, the calcium response would be insensitive to glutamate receptor blockers.

AN-stimulation–evoked calcium responses were quantified for 388 neurons in 14 fields of view from eight animals (Fig. 2*B*,*E*). Of these neurons, 227 were classified as “active” in the control condition. AN-evoked calcium responses in active bushy cells were significantly reduced by bath application of the AMPA receptor antagonist CNQX (control ΔF/F, 1.18 ± 0.08; CNQX, 1.06 ± 0.02; *n* = 106 neurons; Kruskal–Wallis ANOVA; post hoc Dunn's test *p* = 1.04 × 10^−20^) or the combination of CNQX and APV (control, 1.16 ± 0.07; CNQX + APV, 1.04 ± 0.01; *n* = 121 neurons; Dunn's test *p* = 1.14 × 10^−34^). Calcium responses significantly recovered upon wash of glutamate receptor blockers (CNQX recovery, 1.15 ± 0.07; Dunn's test *p* = 2.74 × 10^−13^; CNQX + APV recovery, 1.13 ± 0.06; Dunn's test *p* = 2.54 × 10^−24^; Fig. 2*B–D*). There was a small but significant difference in the ΔF/F values between the CNQX and the CNQX + APV groups (*p* = 0.005), confirming active NMDA receptors in bushy cells (Cao and Oertel, 2010). We explored this difference further by calculating a % suppression for each of the neurons. CNQX suppressed the calcium signal by ∼68% while the cocktail of CNQX + APV suppressed the signal by ∼75% (CNQX: 67.67 ± 12.43%, *n* = 106 neurons; CNQX + APV: 74.51 ± 11.31%, *n* = 121 neurons; Mann–Whitney *U* test; *p* = 0.004; Fig. 2*F*). Across cells, calcium suppression with CNQX and APV was significant but not quite complete, perhaps due to incomplete penetration of antagonists into the thick wedge-slice. However, it is notable that GBCs have a particularly small somatic AP (Oertel, 1983; Cao et al., 2007; Yang et al., 2016) and an abundance of calcium permeable glutamate receptors (Cao and Oertel, 2010). Therefore, the relative contribution of the AP-evoked calcium signal to the total evoked calcium signal may be small, resulting in a relatively large residual calcium signal in the case of incomplete glutamate receptor block even if APs are suppressed. However, the sensitivity of calcium responses in bushy cells to glutamate receptor blockers suggests that AN-stimulation is likely activating CN neurons via synaptic, and not direct electrical excitation, maintaining the AN→CN synapses in the wedge-slice circuit for a more in vivo-like network activation.

### Multicomponent synaptic responses evoked by AN-stimulation

**Figure 3.**
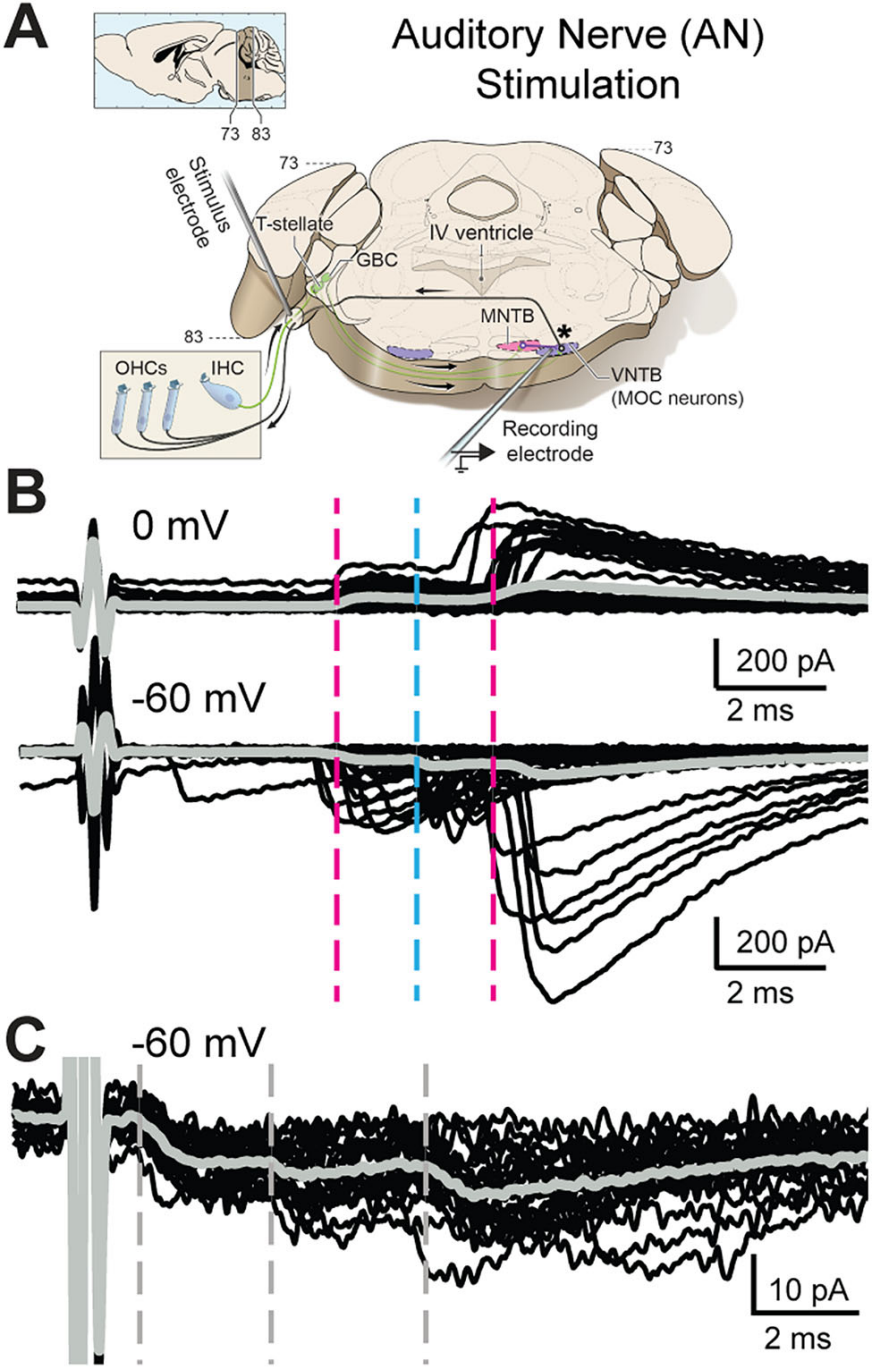
AN-stimulation evokes excitatory and inhibitory PSCs in MOC neurons. ***A***, Schematic of the wedge-slice, indicating electrical stimulation of AN axons (AN-stimulation) projecting to the CN. Schematic by Alan Hoofring, NIH Medical Arts Branch. ***B***, Voltage-clamp traces from identified MOC neurons during AN-stimulation at 1 Hz, 0 mV (top) and −60 mV (bottom). Black traces are 50 overlaid sweeps. Gray line indicates average trace. Dashed lines indicate approximate PSC onset latency for clusters of excitatory (blue) and inhibitory (magenta) PSCs. ***C***, Voltage-clamp traces at −60 mV from a different MOC neuron than in ***B*** of PSCs evoked by AN-stimulation when the stimulus intensity is increased to levels high enough to evoke short-latency PSCs.

To test how the extensive intrinsic circuitry of the intact CN and specialized axons projecting to the SOC change the relative timing of excitatory and inhibitory synaptic inputs to MOC neurons, the AN was electrically stimulated in a wedge-slice while PSCs were recorded in contralateral MOC neurons (Fig. 3*A*). Electrical stimulation at the AN root evoked PSCs in 11 of 43 MOC recordings. Similar to MdL-evoked PSCs, AN-evoked PSCs at a membrane holding potential of −60 mV occurred in multicomponent PSC patterns (Fig. 3*B*). Overall, the latency to the first PSC was significantly longer in AN- versus MdL-evoked PSCs (AN-PSC latency: 5.51 ± 1.00 ms, *n* = 11 cells; MdL-PSC latency: 1.99 ± 0.45 ms; *n* = 21 cells; Mann–Whitney *U* test; *p* = 2.29 × 10^−5^), consistent with an increased total number of intervening synapses causing a longer synaptic delay.

**Figure 4.**
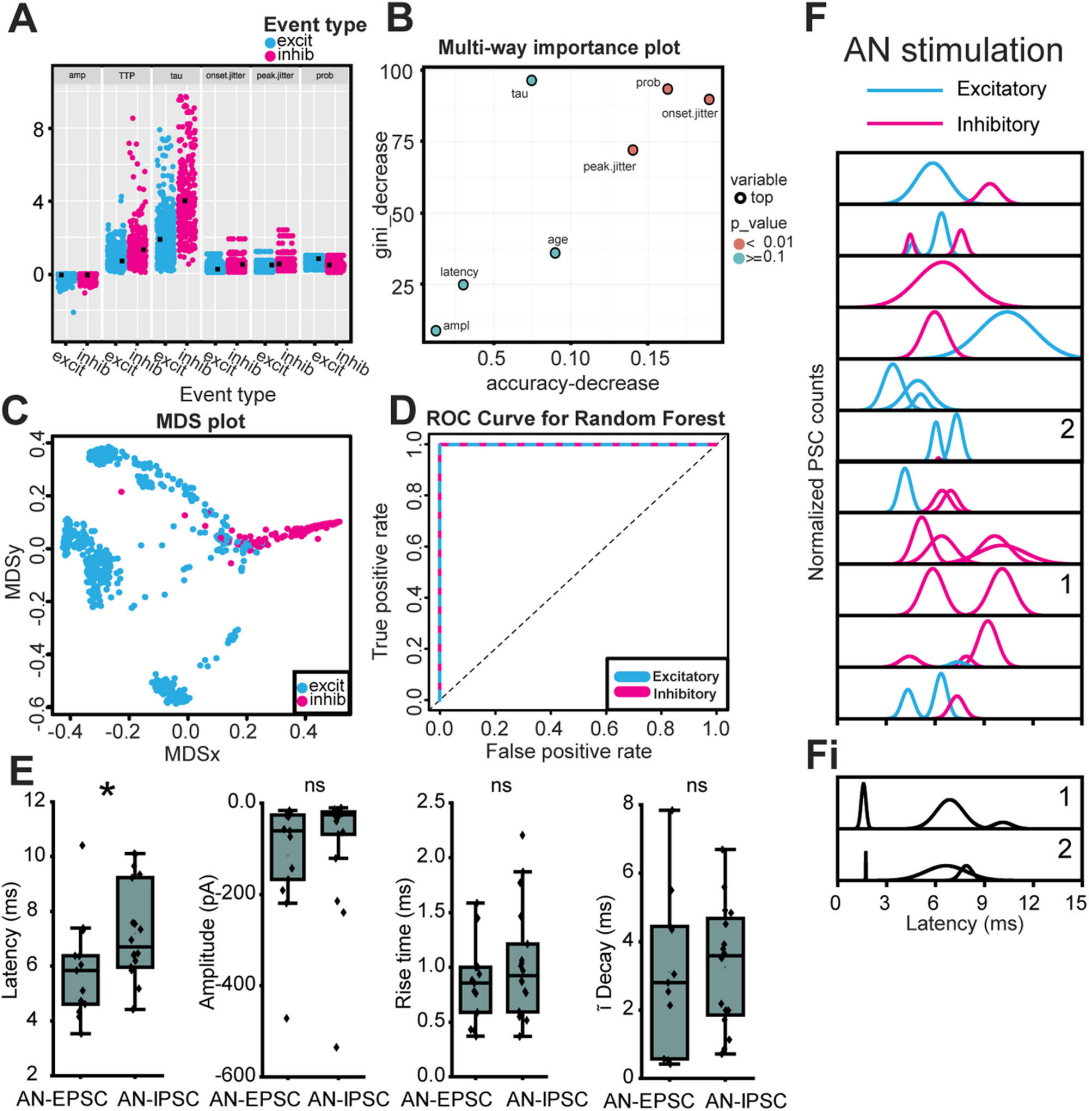
Multicomponent PSCs evoked by AN-stimulation in a wedge-slice. ***A***, Data points for each of seven variables (amp, amplitude; TTP, time to peak; tau, time constant of decay; prob, PSC probability) from MdL-EPSCs (blue) and MdL-IPSCs (magenta) used to train the RandomForest machine learning algorithm. ***B***, Importance plot of variables used for RandomForest algorithm. ***C***, Multidimensional scaling (MDS) plot of proximity matrix showing separation of MdL-EPSCs and MdL-IPSC variables. ***D***, ROC curve for excitatory and inhibitory MdL-PSCs. The algorithm had a 99.89% classification accuracy. ***E***, Comparison of parameters of AN-EPSCs and AN-IPSCs. ***F***, Gaussian fits to frequency histograms (normalized) indicating the latency to clusters of AN-EPSCs (blue) and AN-IPSCs (magenta). Each row represents a different MOC neuron. ***Fi***, Gaussian fits to frequency histograms (normalized) indicating the latency to clusters of AN-PSCs after the stimulation intensity was increased to higher levels that recruit short-latency PSCs. Numbers in panels in ***F*** and ***Fi*** indicate PSC clusters recorded from the same MOC neuron with intermediate (***F***) and high (***Fi***) stimulation intensities.

In two experiments, AN-stimulation protocols were repeated with high intensity electrical stimulation (>2,000 μA), to cause greater current spread in the tissue and intentionally bypass the AN→CN synapse. The high stimulation intensity significantly decreased PSC latency (AN-high stim level latency: 1.65 ± 0.1 ms, *n* = 18 PSCs; AN-intermediate stim level latency: 4.58 ± 0.3 ms, *n* = 27 PSCs; *n* = 2 neurons; Mann–Whitney *U* test; *p* = 1.90 × 10^−8^; Figs. 3*C*, 4*Fi*). This indicates that with intentionally high stimulation intensity, CN axons projecting to MOC neurons were directly stimulated, bypassing intrinsic circuitry of the CN. The remaining experiments used the lower stimulation intensity that synaptically engages, not electrically bypasses, the full complement of CN circuits.

### Machine learning algorithm to classify excitatory and inhibitory PSCs

Analysis of synaptic integration requires classification of synaptic responses as excitatory or inhibitory. In some (4/11) experiments with AN-stimulation, the MOC neuron membrane holding potential was set to 0 mV to isolate IPSCs. Similar to MdL-stimulation recordings, IPSCs occurred in clusters that aligned with a subset of the clusters recorded at −60 mV (Fig. 3*B*), indicating that both EPSCs and IPSCs are evoked in MOC neurons by AN-stimulation. However, for the cells lacking 0 mV data, we were initially unable to classify PSC clusters. Therefore, we used parameters of PSCs evoked from MdL-stimulation to develop a machine learning approach to characterize EPSCs and IPSCs and then used this algorithm of MdL-evoked PSC characteristics to classify AN-evoked PSCs as excitatory or inhibitory. To accomplish this, first we developed a RandomForest-based classification algorithm to identify EPSCs and IPSCs by training the model on identified MdL-EPSCs and MdL-IPSCs recorded at −60 mV (Fig. 4*A–D*).

Next, we used the trained RandomForest algorithm to classify AN-PSCs recorded at −60 mV as excitatory or inhibitory. The 344 AN-PSCs from 11 MOC neuron recordings were clustered in the same manner as the MdL-PSCs (above), and the PSC parameters were calculated on a PSC-by-PSC basis (rise time, time constant of decay, amplitude, animal age) or a cluster-by-cluster basis (onset jitter, peak jitter, PSC probability). The AN-PSCs were then fed into the trained RandomForest algorithm, which gave the probability that each AN-PSC could be classified as AN-EPSC or AN-IPSC.

### AN-evoked synaptic responses

We then determined the effect of AN-PSCs on MOC neurons. Following classification of AN-EPSCs and AN-IPSCs responses at −60 mV using the RandomForest algorithm above, the parameters of AN-EPSCs and AN-IPSCs for all clusters were summarized to determine their effects on MOC neurons (Fig. 4*E*). AN-EPSCs and AN-IPSCs in all clusters at −60 mV had similar amplitudes (AN-EPSC amplitude: 60.6 ± 39.0 pA, *n* = 13 clusters, *n* = 8 cells; AN-IPSC amplitude: 25.6 ± 11.1 pA, *n* = 17 clusters, *n* = 10 cells; Mann–Whitney *U* test; *p* = 0.14) and kinetics (AN-EPSC rise time: 0.86 ± 0.15 ms, *n* = 13 clusters, *n* = 8 cells; AN-IPSC rise time: 0.92 ± 0.31 ms, *n* = 17 clusters, *n* = 10 cells; Mann–Whitney *U* test; *p* = 0.35; AN-EPSC time constant of decay: 2.80 ± 1.65 ms, *n* = 11 clusters, *n* = 8 cells; AN-IPSC time constant of decay: 3.59 ± 1.50 ms, *n* = 16 clusters, *n* = 10 cells; Mann–Whitney *U* test; *p* = 0.67). The median AN-EPSC latency occurred with a significantly shorter latency than AN-IPSCs (AN-EPSC latency: 5.83 ± 1.22 ms, *n* = 13 clusters, *n* = 8 cells; AN-IPSC latency: 6.71 ± 0.87 ms, *n* = 18 clusters, *n* = 10 cells; Mann–Whitney *U* test; *p* = 0.04). AN-evoked PSCs also occurred in complicated patterns that could have multiple excitatory or inhibitory clusters (Fig. 4*F*, each peak indicates a different “cluster”). PSCs evoked by AN-stimulation were more likely to have “complex” patterns with two or more clusters of either EPSCs or IPSCs (AN 8/11 vs MdL 8/21 complex clusters), interpreted here as PSCs from multiple presynaptic sources.

**Figure 5.**
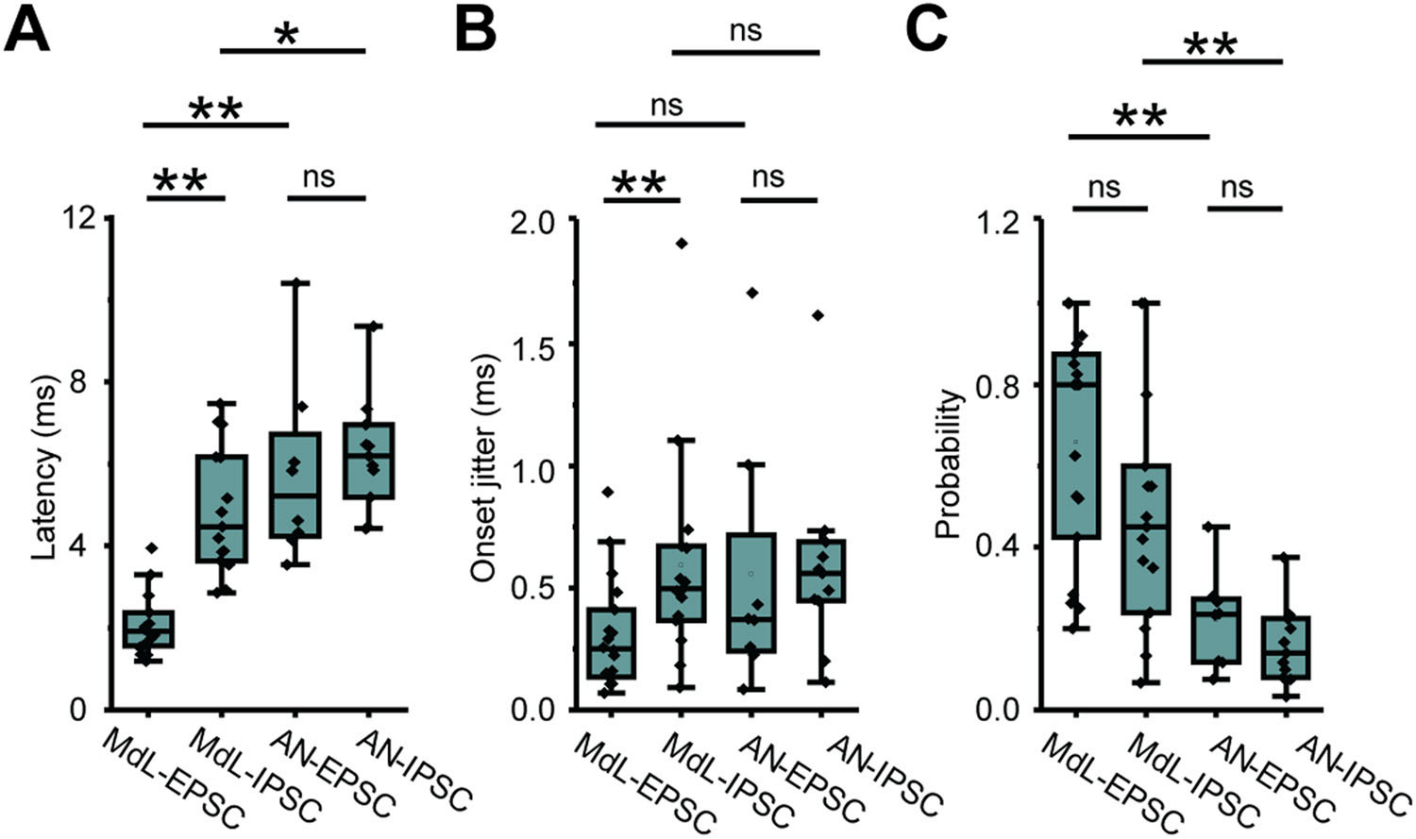
Comparison of PSCs evoked from MdL- versus AN-stimulation. ***A–C***, Plot of ***A***, latency to PSC onset, ***B***, synaptic jitter of PSC onset, and ***C***, probability of recording a PSC for MdL- versus AN-EPSCs and IPSCs in the first cluster recorded in each MOC neuron.

Comparison of PSCs evoked by MdL- versus AN-stimulation demonstrated a specialized function of CN circuitry, which was both intact and engaged during AN-stimulation but not MdL-stimulation. Figure 4 examines all AN-PSCs, but here we consider the first excitatory cluster and first inhibitory cluster to isolate our analyses to direct ascending pathways. The overall latency to the first AN-PSCs was increased compared with MdL-PSCs, as expected because AN-stimulation includes at least one more synapse (AN→CN neurons) compared with MdL-stimulation. This overall increase in latency with AN- compared with MdL-stimulation occurred for both EPSCs and IPSCs (MdL-EPSC latency: 1.92 ± 0.37 ms, *n* = 18 clusters, *n* = 18 cells; AN-EPSC latency: 5.22 ± 0.98 ms, *n* = 8 clusters, *n* = 8 cells; Mann–Whitney *U* test; *p* = 8.98 × 10^−5^; MdL-IPSC latency: 4.47 ± 0.93 ms, *n* = 15 clusters, *n* = 15 cells; AN-IPSC latency: 6.19 ± 0.77 ms, *n* = 11 clusters, *n* = 11 cells; Mann–Whitney *U* test; *p* = 0.049). However, the additional circuitry added during AN-stimulation does not alter EPSC and IPSC timing to the same degree. When comparing MdL-stimulation evoked EPSCs and IPSCs, MdL-EPSCs are significantly shorter latency than MdL-IPSCs, and MdL-IPSCs are never recorded before MdL-EPSCs in any cell (MdL-EPSC latency: 1.92 ± 0.37 ms, *n* = 18 clusters, *n* = 18 cells; MdL-IPSC latency: 4.47 ± 0.93 ms, *n* = 15 clusters, *n* = 15 cells; Mann–Whitney *U* test; *p* = 4.79 × 10^−6^). In contrast, when stimulating the AN, there is no significant difference in latency between AN-EPSCs and AN-IPSCs (AN-EPSC latency: 5.22 ± 0.98 ms, *n* = 8 clusters, *n* = 8 cells; AN-IPSC latency: 6.19 ± 0.77 ms, *n* = 11 clusters, *n* = 11 cells; Mann–Whitney *U* test; *p* = 0.30), suggesting that the CN circuitry can compensate in speed for the additional synapse present in the IPSC pathway to equalize the timing of the excitatory and inhibitory pathways (Fig. 5*A*). This is also apparent when calculating the “E–I latency difference” on a cell-by-cell basis, for which the latency to the first IPSC is subtracted from the latency to the first EPSC. MdL-stimulated EPSCs all had positive E–I latency difference values that were significantly different from zero (one-sample Wilcoxon signed-rank test; *p* = 0.0039), indicating that excitation always preceded inhibition. However, the AN-stimulation E–I latency difference was smaller than the MdL-stimulation E–I latency difference (MdL: 2.80 ± 0.88, *n* = 11 cells; AN: 0.14 ± 2.87, *n* = 7 cells; Mann–Whitney *U* test; *p* = 0.085), and not significantly different from zero (one-sample Wilcoxon signed-rank test; *p* = 1). Further, two AN-stimulation experiments had negative E–I latency differences, indicating that inhibition preceded excitation in these cells, similar to projections to MSO neurons (Roberts et al., 2013). These data confirm the unusual speed of the inhibitory pathway from the CN to the SOC relative to the excitatory pathway. Further, comparison of AN-stimulation and MdL-stimulation synaptic timing demonstrates that the remarkable speed of the inhibitory pathway is localized to components of the circuit added to the preparation during AN-stimulation, namely, the AN synapse onto the GBCs and the GBCs themselves.

Auditory brainstem circuits contain extremely precise neurons, so the timing and precision of synaptic inputs evoked from MdL- versus AN-stimulation experiments was investigated to determine if MOC synaptic inputs are comparably temporally precise. The onset jitter of PSCs was computed for the first EPSC cluster and first IPSC cluster per MOC neuron for both MdL- and AN-stimulation (Fig. 5*B*). A simplistic view suggests that each additional synapse in a pathway would increase the overall pathway jitter, resulting in EPSCs having less jitter than IPSCs in all recording configurations. Indeed, monosynaptic T-stellate→MOC MdL-EPSCs had lower jitter than disynaptic GBC→MNTB→MOC MdL-IPSCs (MdL-EPSC jitter: 0.25 ± 0.13 ms, *n* = 18 clusters, *n* = 18 cells; MdL-IPSC jitter: 0.50 ± 0.17 ms, *n* = 15 clusters, *n* = 15 cells; Mann–Whitney *U* test; *p* = 0.015). However, AN-EPSC jitter is not different from AN-IPSC jitter (AN-EPSC jitter: 0.37 ± 0.13 ms, *n* = 8 clusters, *n* = 8 cells; AN-IPSC jitter: 0.56 ± 0.11 ms, *n* = 11 clusters, *n* = 11 cells; Mann–Whitney *U* test; *p* = 0.30). The lack of difference in jitter between the excitatory and inhibitory pathways with AN-stimulation suggests that the inhibitory pathway can compensate for having an additional synapse by having enhanced precision in the CN. There was no difference in PSC probability between MdL-EPSCs and MdL-IPSCs (MdL-EPSC probability: 0.80 ± 0.19, *n* = 18 clusters, *n* = 18 cells; MdL-IPSC probability: 0.45 ± 0.15, *n* = 15 clusters, *n* = 15 cells; Mann–Whitney *U* test; *p* = 0.08), and there was also no difference in probability of AN-EPSCs and AN-IPSCs (AN-EPSC probability: 0.24 ± 0.08, *n* = 8 clusters, *n* = 8 cells; AN-IPSC probability: 0.14 ± 0.06, *n* = 11 clusters, *n* = 11 cells; Mann–Whitney *U* test; *p* = 0.16). However, when stimulating at the AN, both the excitatory and inhibitory pathways have a lower probability of PSCs compared with stimulating at the MdL (MdL-EPSC vs AN-EPSC: Mann–Whitney *U* test; *p* = 0.00114; MdL-IPSC vs AN-IPSC: Mann–Whitney *U* test; *p* = 0.002; Fig. 5*C*). The inhibitory pathway has a high proportion of suprathreshold PSPs in the CN (Oertel, 1983; Smith and Rhode, 1987; Cao and Oertel, 2010). Therefore, these results could indicate a reduced throughput at the endbulb→GBC synapse compared with stimulating the GBC→MNTB synapse alone in our experiments with MdL-stimulation or could be a result of severing axons from the CN during the slicing procedure that in turn reduces the numbers of activated MNTB neurons that converge onto MOC neurons.

### Summation of ascending MdL-evoked synaptic inputs drives APs in MOC neurons

**Figure 6.**
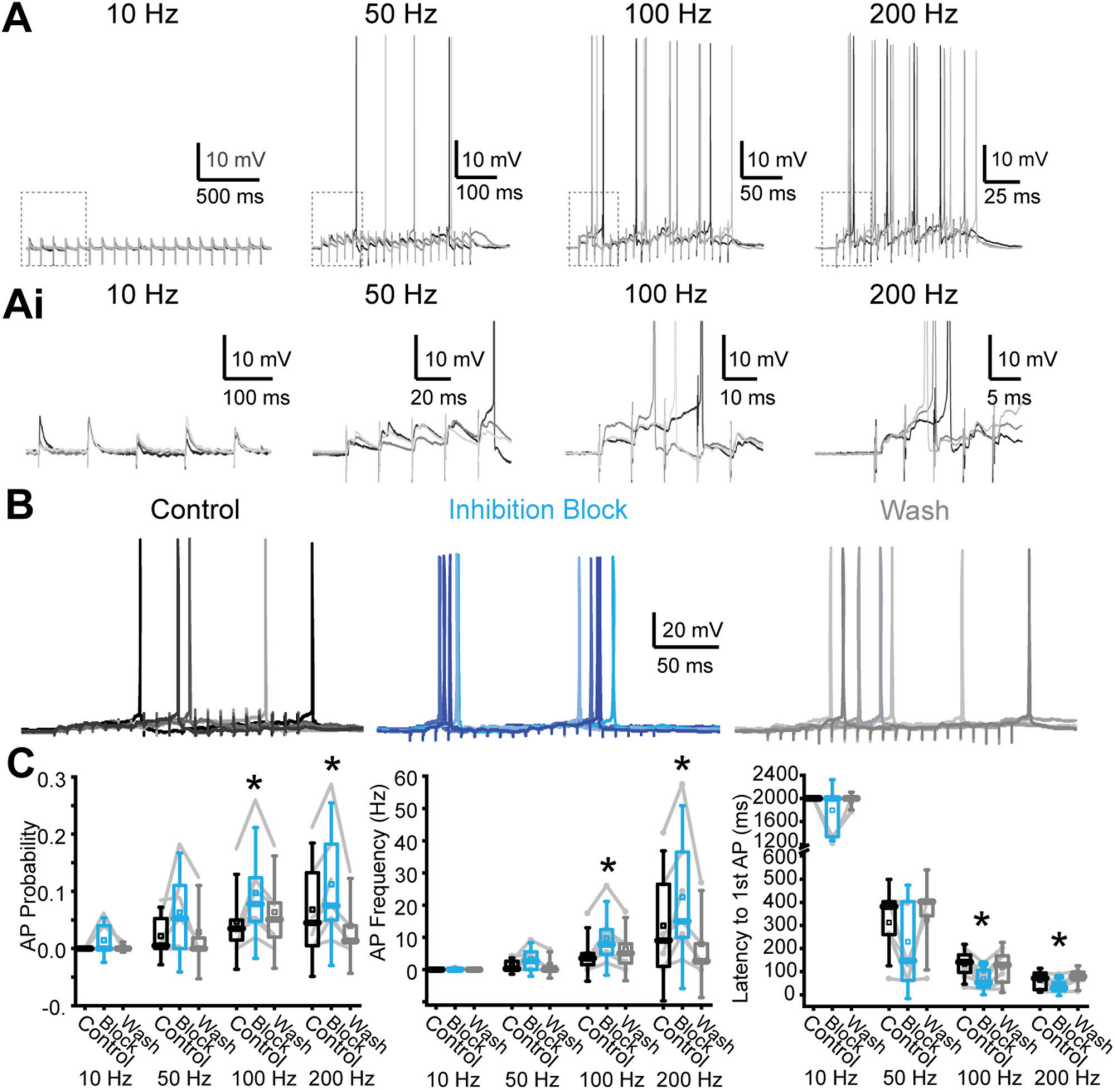
Inhibition reduces AP activity in MOC neuron recordings in response to MdL-PSPs. ***A***, Current-clamp recordings from an MOC neuron while evoking MdL-PSPs for 20 pulses at 10, 50, 100, and 200 Hz. Three sweeps shown, overlaid, at each stimulation rate. ***Ai***, Zoom of regions in each panel of ***A*** indicated by dashed box, showing the first five PSPs evoked in MdL-stimulation trains. ***B***, 100 Hz MdL-stimulation–evoked PSPs summate to generate AP trains in control conditions (left), in the presence of 50 µM gabazine and 1 µM strychnine to block inhibitory inputs (“Inhibition block,” center), and after wash of inhibitory receptor blockers (right). Five sweeps are overlaid per condition. ***C***, Quantification of AP probability (left), frequency (center), and latency to first AP (right) for 5–8 neurons.

The voltage-clamp experiments above demonstrate that the inhibitory pathways projecting to MOC neurons can be remarkably fast when the in vivo-like circuitry is intact. However, the impact of the timing of inhibition on MOC neuron AP activity is unknown. We tested integration under more physiological conditions of low intracellular chloride (7.2 mM; *E*_Cl _= −74 mV), without intracellular QX-314, and in the current-clamp configuration. We stimulated both excitatory and inhibitory synaptic pathways simultaneously to record PSPs and resulting APs in MOC neurons using the MdL-stimulation configuration because these experiments were high-throughput enough to allow pharmacological blockade of inhibitory synaptic inputs. MdL-stimulation was applied in trains to evoke PSPs (MdL-PSPs; Fig. 6*A*,*Ai*). Summation of MdL-PSPs evoked APs, with increased stimulation rates evoking APs with an increased probability, increased rate, and reduced latency to the first AP (Friedman ANOVA: AP probability, *p* = 0.031; AP rate, *p* = 0.007; number of stimulations to first AP, *p* = 0.041; latency to first AP, *p* = 4.6 × 10^−4^; Table 3; Fig. 6*A*,*C*).

**Table 3.** Measurements of AP probability, rate, the number of stimulations to first AP, and latency to first AP in 5–8 MOC neuron recordings in response to MdL-stimulation at the frequency indicated, from Figure 6*C*

Rate	Treatment (n)	AP Prob	AP rate (Hz)	# stimulations to first AP	Latency to first AP
10 Hz	Control (7)	0	0	>20	>2,000
10 Hz	Block (7)	0	0	>20	>2,000
10 Hz	Wash (5)	0	0	>20	>2,000
50 Hz	Control (7)	0.005 ± 0.005	0.25 ± 0.25	19.0 ± 1.0	379.2 ± 20.9
50 Hz	Block (7)	0.053 ± 0.053	2.63 ± 2.63	7.80 ± 4.40	144.4 ± 86.87
50 Hz	Wash (5)	0	0	>20.0	>400
100 Hz	Control (7)	0.035 ± 0.015	3.49 ± 1.51	14.15 ± 4.30	139 ± 44.6^a^
100 Hz	Block (7)	0.078 ± 0.036*	7.78 ± 3.75*	5.40 ± 2.76*	49.8 ± 29.1*
100 Hz	Wash (5)	0.051 ± 0.03	5.08 ± 3.00	9.50 ± 5.13	126 ± 56.7
200 Hz	Control (7)	0.045 ± 0.040^a^	9.00 ± 8.00^a^	14.2 ± 4.20^a^	69.3 ± 22.3^a^
200 Hz	Block (7)	0.075 ± 0.047*	15.0 ± 9.40*	6.00 ± 2.00*	26.3 ± 12.0*
200 Hz	Wash (5)	0.013 ± 0.013	2.67 ± 2.67	16.6 ± 3.50	75.7 ± 19.5

Measurements are presented in control, during pharmacological blockade of postsynaptic inhibitory receptors (“block”), and wash conditions, as median ± MAD. Numbers in parentheses indicate *n*.

a Indicates significantly different from control 10 Hz stimulation (Friedman ANOVA with post hoc Dunn's test).

* Indicates significantly different from control within stimulus frequency comparison (*p* < 0.05, paired Wilcoxon signed-rank test).

We then tested the effect of inhibition on AP rates in MOC neurons in response to MdL-stimulation by blocking inhibitory neurotransmitter receptors. In the absence of inhibitory neurotransmission, there was an increased AP probability and rate, decreased number of stimulations to the first AP, and decreased latency to the first AP for 100 and 200 Hz stimulation rates (Table 3; Fig. 6*B*,*C*).

### Computational model of MOC neurons to assess integration of excitation and inhibition

**Figure 7.**
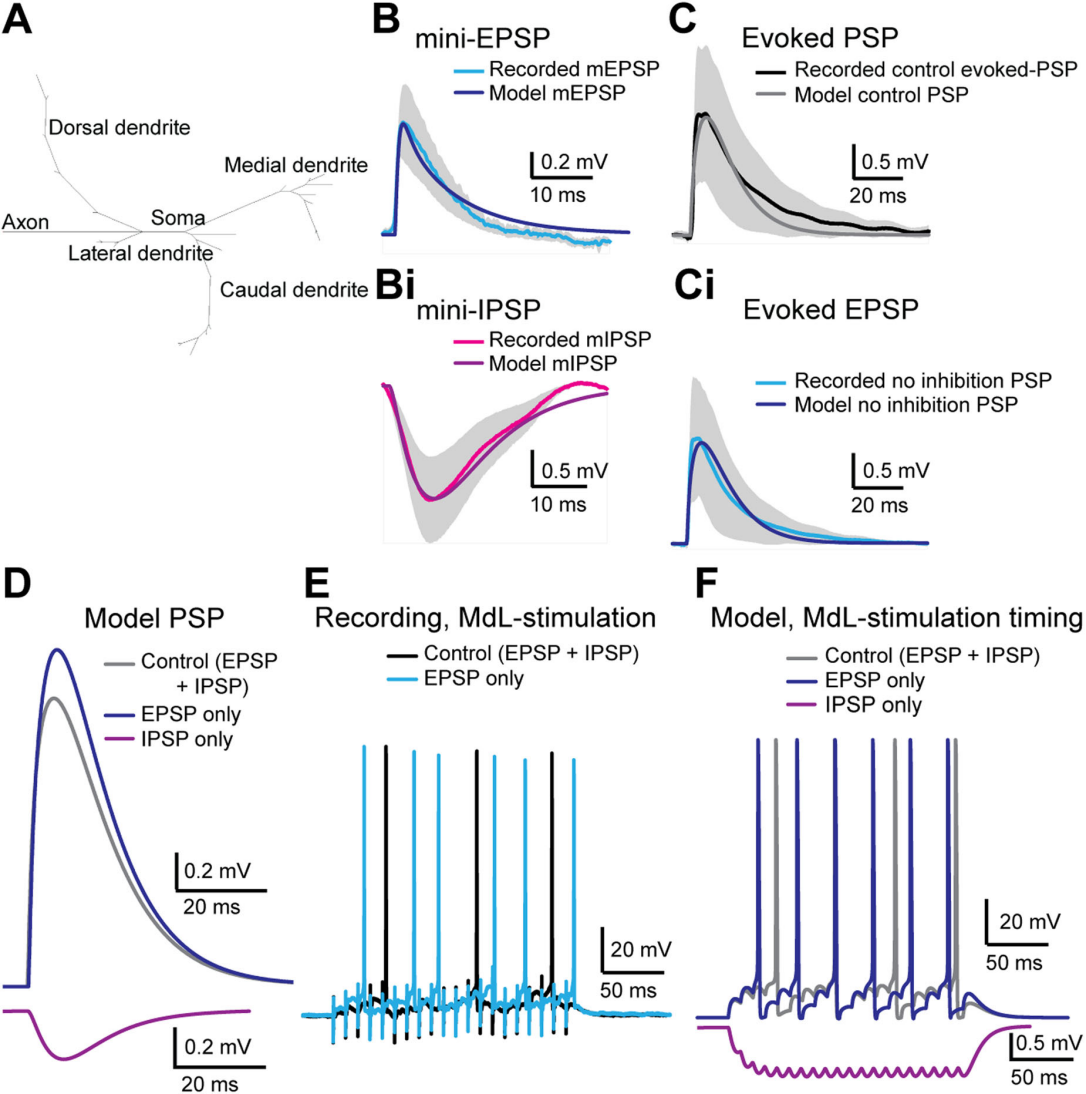
A computational MOC model replicates responses to MdL-stimulation. ***A***, Topology of the model MOC neuron. ***B***, Waveform of mEPSPs from MOC neuron recordings (light blue line, gray shading indicates standard deviation) with overlaid mEPSP generated from the model MOC neuron (dark blue). ***Bi***, Waveform of mIPSPs from MOC neuron recordings (magenta line, gray shading indicates standard deviation) with an overlaid mIPSP generated from the model MOC neuron (purple). ***C***, Waveform of control evoked PSPs from MOC neuron recordings (combined EPSP and IPSP, black line, gray shading indicates standard deviation) overlaid with PSPs from the model MOC neuron in control conditions (combined EPSP and IPSP, gray line). ***Ci***, Waveform of excitatory evoked-PSP from MOC neuron recordings after pharmacological block of inhibitory receptors (no inhibition, EPSP only, blue line, gray shading indicates standard deviation) overlaid with PSPs from the model MOC neuron with excitatory PSPs only (no inhibition, EPSP only, dark blue line). ***D***, Comparison of model evoked PSPs in control (EPSP and IPSP, gray), excitatory only (inhibition removed, EPSP only, dark blue), and inhibitory only conditions (excitation removed, IPSP only, purple). ***E***, Current-clamp recordings from MOC neurons during 100 Hz, 20 pulse MdL-stimulation to evoke APs in control (evoked EPSP and IPSP, black) and excitatory only conditions (inhibition blocked, EPSP only, blue). ***F***, Response of the model MOC neuron to PSPs simulated at 100 Hz, 20 pulse trains with synaptic timing mimicking MdL-stimulation synaptic timing in control (EPSP and IPSP, gray), excitatory only (inhibition removed, EPSPs only, dark blue), and inhibitory only conditions (excitation removed, IPSP only, purple).

In the MdL-stimulation experiments above, pharmacological blockade of inhibition increased APs in MOC neurons. We next asked how the effect of inhibition on MOC activity would change when excitatory and inhibitory pathways were stimulated with the in vivo-like synaptic timing that we observed in the AN-stimulation experiments. However, the short window of time for wedge-slice viability for AN-stimulation experiments made the additional pharmacological experiments necessary for testing the effect of inhibition on MOC neuron activity prohibitive. Therefore, we built a computational model of an MOC neuron to test the integration of excitation and inhibition with synaptic inputs that mimic AN-stimulation experiments. The model MOC neuron had topology generated from a published MOC neuron morphology [Brown and Levine (2008), their Fig. 9; Fig. 7*A*], and ion channels and biophysical properties were tuned to mimic the recorded synaptic and AP activity of MOC neurons (Materials and Methods).

The model MOC neuron responded to synaptic inputs based on recorded mini-EPSPs (mEPSP) and mini-IPSPs (mIPSP) with closely matched amplitudes and waveforms (Fig. 7*B*,*Bi*), confirming that the model MOC neuron responds to synaptic inputs similarly to biological MOC neurons. Next, synaptic potentials were simulated within the model MOC neuron to mimic PSPs evoked from MdL-stimulation experiments both in control conditions and with pharmacological blockade of inhibitory synaptic inputs (Fig. 7*C*,*Ci*). IPSPs were then added to the model to mimic MdL-stimulation conditions, with the IPSP onset being longer latency than the EPSP onset by +2.8 ms (E–I latency difference +2.8 ms), as measured in MOC neuron MdL-stimulation recordings (Fig. 7*C*,*D*).

The responses of the model MOC neuron to repeated synaptic inputs were then tested by comparing model responses to activity recorded in MOC neurons in response to 100 Hz trains of MdL-stimulation (Figs. 6, 7*E*). Trains of MdL-PSPs were simulated within the model, first using the relative timing of excitatory and inhibitory synaptic inputs recorded during MdL-stimulation as above (E–I latency difference: +2.8 ms) and then with IPSPs absent to mimic pharmacological blockade of IPSPs in MOC neuron recordings (Fig. 7*F*). Consistent with electrophysiology data, single control PSPs (EPSPs + IPSPs) did not evoke APs in the model MOC neuron. However, summation of PSPs evoked APs (latency 38.5 ms), similar to the latency to AP in MdL-stimulation experiments (49.75 ± 29.06 ms). IPSPs were then removed from the model and summation of EPSPs initiated APs with a shorter latency (24.4 ms). This decrease in latency to APs in the absence of IPSPs is consistent with recordings from MOC neurons and consistent with inhibition delaying APs.

**Figure 8.**
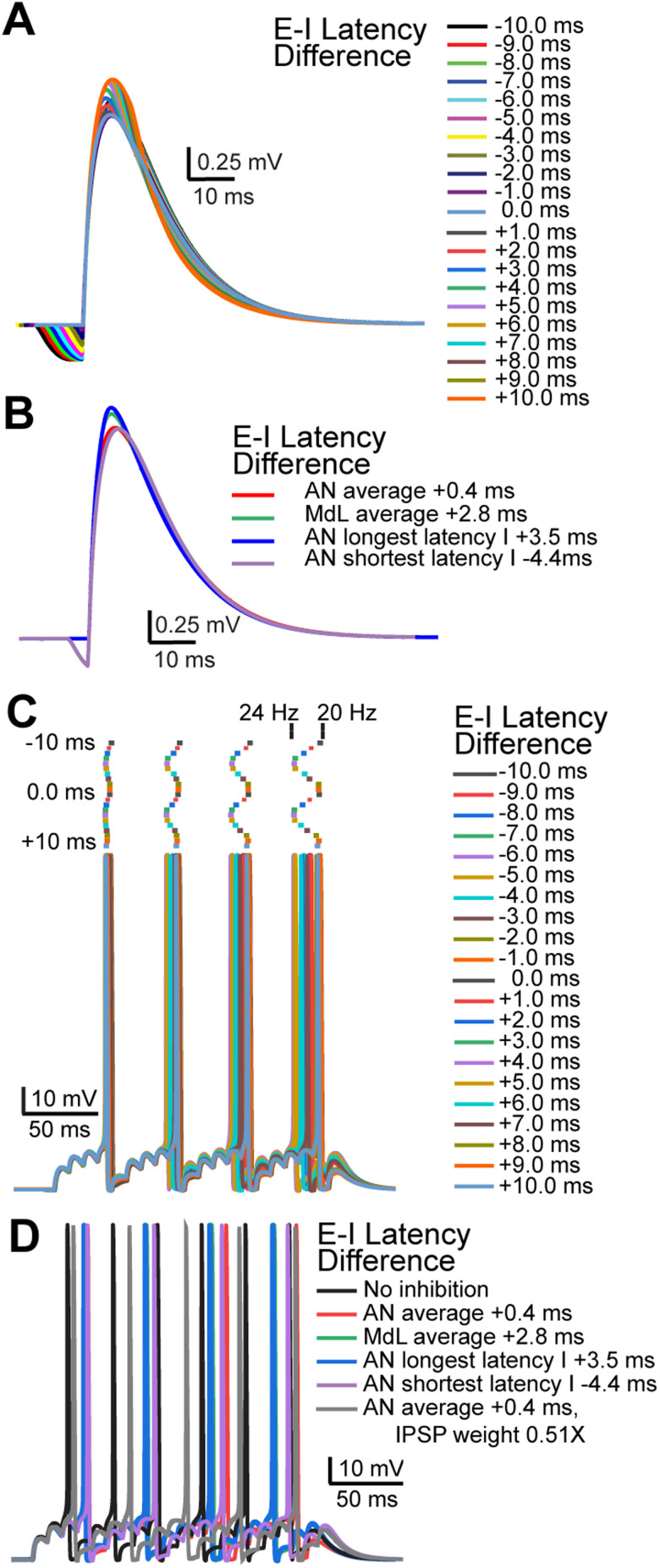
Varying latencies of summating EPSPs and IPSPs adjust model MOC neuron AP onset and rate. ***A***, PSPs in the model MOC neuron resulting from summation of EPSPs and IPSPs at the E–I latency differences indicated. ***B***, PSPs in the model MOC neuron resulting from summation of EPSPs and IPSPs at E–I latency differences from recorded MOC neurons. ***C***, APs in the model MOC neuron resulting from summation of 100 Hz trains of simulated EPSPs and IPSPs at the E–I latency differences indicated. Raster plots above the APs indicate timing of AP peak. Changing E–I latency differences adjusted the AP rate between ∼20 and 24 Hz, indicated above the rasters. ***D***, APs in the model MOC neuron resulting from summation of simulated EPSPs and IPSPs with E–I latency differences recorded in MOC neurons or with reduced a IPSP amplitude (0.51 times the control value) to mimic prior synaptic depression.

We next used the MOC neuron model to determine how the timing of inhibition relative to excitation affects MOC neuron activity in the in vivo*-*like AN-stimulation configuration. In this configuration, the median latency to synaptic inhibition is closer to that of synaptic excitation compared with the greater synaptic timing separation in the MdL-stimulation configuration and in some cells, inhibition preceded excitation (Figs. 3, 4). We first generated single PSPs with varying excitatory and inhibitory synaptic timing ranging from EPSPs preceding IPSPs from 0 to 10 ms in 1 ms increments (E–I latency difference 0 to +10 ms), and IPSPs preceding EPSPs from 0 to 10 ms in 1 ms increments (E–I latency difference 0 to −10 ms). Integrated PSPs generated from these combinations had systematically varying amplitudes (Fig. 8*A*). Simulating PSPs with the most extreme observed E–I latencies from AN-stimulation recordings also resulting in integrated PSPs with varying amplitudes (Fig. 8*B*).

We then simulated trains of PSPs in the MOC model to analyze the effect of synaptic timing on AP latency and rate. PSPs with E–I latencies from −10 to +10 ms were simulated at rates of 100 Hz. PSPs summated to evoke APs with latencies and rates that systematically varied depending on E–I latency difference (Fig. 8*C*). With only EPSPs (IPSPs removed), model APs occurred at 31.5 Hz. Adding simulated inhibitory PSPs during train stimulation increased the latency to the first AP and reduced AP rate in all cases. The degree of the effect of inhibition depended on the E–I latency difference, with the most effective inhibition occurring with E–I latency differences of −10, −1, and +9 ms (AP latency, ∼48 ms; AP rate, ∼20 Hz) and the least effective inhibition occurring with E–I latency differences of −6.0 and +4.0 ms (AP latency, ∼43 ms; AP rate, ∼23.5 Hz). In wedge-slice recordings with AN-stimulation, E–I latency differences in all cells ranged from −4.4 to +3.5 ms, a range that nearly includes both minimal and maximal effects of inhibition on increasing AP latency and decreasing AP rate. Notably, the median AN-evoked E–I latency difference (+0.4 ms) had a greater effect on delaying and decreasing the rate of APs compared with the median MdL-evoked E–I latency difference (+2.8 ms; Fig. 8*D*), indicating that inhibitory synaptic inputs have a greater effect on suppressing APs in MOC neurons when occurring with the in vivo-like timing measured in AN-stimulation experiments. However, there was a much reduced effect of inhibition if the IPSP amplitude was decreased to match recordings in which background MNTB activity caused synaptic depression of IPSPs (Torres Cadenas et al., 2022; Fig. 8*D*). The variability in timing of inhibitory inputs recorded in different MOC neurons paired with the degree of prior synaptic depression suggests that inhibition may have a different effect on each MOC neuron in the brain and can thus flexibly adjust MOC neuron activity and desynchronize the population of MOC neurons.

Together, our results indicate that inhibition from the MNTB can delay initial spiking of MOC neurons and that the precise timing of inhibition can flexibly adjust MOC activity rates. In addition, the inhibitory pathway to MOC neurons is exceptionally fast and precise, due to the properties of synaptic transmission and neuron function localized to the AN synapses onto CN cells, the CN cells themselves, and CN projection axons.

## Discussion

Diverse synaptic inputs to MOC neurons likely converge to adjust MOC function under changing hearing conditions. The responses of MOC neurons in vivo have low thresholds, V-shaped tuning curves, sound-evoked firing rates up to 110 Hz, and a variable AP latency from sound onset that decreases with loud sounds in guinea pig (Robertson and Gummer, 1985; Brown, 1989) and cat (Fex, 1962; Liberman and Brown, 1986; Liberman, 1988). Histological and lesion experiments described synaptic inputs to MOC neurons from a variety of auditory and nonauditory neurons in rat (Faye-Lund, 1986; Caicedo and Herbert, 1993; Vetter et al., 1993; Mulders and Robertson, 2000, 2002; Groff and Liberman, 2003; Gómez-Nieto et al., 2008), mouse (Darrow et al., 2012; Suthakar and Ryugo, 2017), and guinea pig (Thompson and Thompson, 1993; Horvath et al., 2003; Ota et al., 2004; De Venecia et al., 2005; Benson and Brown, 2006; Brown et al., 2013). In vivo extracellular recordings and tract tracing studies suggest possible excitatory synaptic inputs from CN SCC neurons in cat (Ye et al., 2000) and guinea pig (Hockley et al., 2022). In vitro patch-clamp recordings of synapses to MOC neurons include functional demonstration of ascending excitation from CN T-stellate cells, and descending, facilitating excitation from the IC in mice (Romero and Trussell, 2021). Inhibitory synaptic inputs were recorded in putative rat MOC neurons (Robertson, 1996) and more recently originating in the MNTB as shown in genetically identified MOC neurons in mouse (Torres Cadenas et al., 2020). Serotonergic excitation of mouse MOC neurons (Suthakar and Weisz, 2023) suggests modulatory inputs. These diverse inputs likely underestimate the convergence of synapses to MOC neurons. Our in vitro wedge-slice preparation combined with machine learning and single-neuron computational modeling is a first step in probing synaptic integration in MOC neurons in vitro, from monaural ascending circuits, with in vivo-like timing.

### Functional synaptic inputs to MOC neurons

The wedge-slice includes monaural ascending circuitry to MOC neurons beginning with AN axons from Type I SGN onto CN neurons. CN axons to the SOC are intact (Fischl and Weisz, 2020). Type II SGN terminations in the CN including onto granule and other cells (mouse: Berglund et al., 1996; Benson and Brown, 2004; Weisz et al., 2021) are also present. Intrinsic CN circuitry remains intact, including excitation and inhibition (reviewed in Cant and Benson, 2003; Kuenzel, 2019; mouse: Wickesberg and Oertel, 1988; Ferragamo et al., 1998; Xie and Manis, 2013, 2014; Campagnola and Manis, 2014; Muniak and Ryugo, 2014; Ngodup et al., 2020; rat: Doucet and Ryugo, 1997; guinea pig: Arnott et al., 2004; and cat: Rhode and Greenberg, 1994; Ostapoff et al., 1999) and reciprocal connectivity within bushy (rat: Gómez-Nieto and Rubio, 2009) and T-stellate cells (mouse: Cao et al., 2019). Absent from the preparation are the cochlea, commissural CN pathways, and most descending circuits to the CN. The varied patterns of multiple PSC peaks, found both in AN- and MdL-stimulation experiments, suggest diverse excitatory and inhibitory inputs. These patterns suggest either additional direct CN projections or polysynaptic inputs with a contralateral CN origin. Additional potential sources of excitation are SCC neurons in cat (Ye et al., 2000) and guinea pig (Hockley et al., 2022), axon collaterals from GBCs (bats and rodents: Kuwabara et al., 1991; cat: Smith et al., 1991), or other as-yet unidentified sources. For inhibition, there may be a direct projection from the CN (mouse: Weingarten et al., 2023), but polysynaptic inputs are likely because of the ∼3–4 ms delay between IPSC clusters in most recordings. Potential polysynaptic inhibitory inputs include from the LNTB (gerbil: Cant and Hyson, 1992; bats and rodents: Kuwabara and Zook, 1992), the superior peri-olivary nucleus (SPON) in rat (Kelly et al., 1998; Kulesza and Berrebi, 2000), other inhibitory VNTB neurons in mouse (Albrecht et al., 2014), or the MOC neurons themselves, which are cholinergic but likely also GABAergic in mouse (Wedemeyer et al., 2013; Kitcher et al., 2022; Frank et al., 2023; Bachman et al., 2024).

### Speed and fidelity of the inhibitory pathway

The GBC→MNTB pathway inhibits other SOC neurons, including the LSO, MSO, lateral olivocochlear (LOC), and SPON neurons. The pathway has large endbulb of Held synapses from the AN onto GBCs, while GBCs have large-diameter, heavily myelinated axons (cat: Warr, 1972; Brownell, 1975; mouse: Lauer et al., 2013; guinea pig: Ford et al., 2015) and terminate in the large calyx of Held onto MNTB neurons. High-fidelity synapses throughout the pathway allow activity up to hundreds of Hz in cat (Guinan et al., 1972; Brownell, 1975; Guinan and Li, 1990; Spirou et al., 1990; Smith et al., 1998; Mc Laughlin et al., 2014), rat (Sommer et al., 1993; Borst et al., 1995; Takahashi et al., 1996; Taschenberger and von Gersdorff, 2000; Tolnai et al., 2008), mouse (Wu and Kelly, 1993; Barnes-Davies and Forsythe, 1995), and gerbil (Kopp-Scheinpflug et al., 2003, 2008, 2011; Englitz et al., 2009). The high speed of this pathway was demonstrated in the GBC→MNTB synapses to the MSO in gerbil (Roberts et al., 2013). In our present results in mice, in two MOC neuron recordings the inhibitory pathway is shorter latency than the excitatory pathway, despite an additional synapse, indicating that even while lacking some axonal GBC specializations present in gerbils (Stange-Marten et al., 2017), mice also have an exceptionally fast GBC→MNTB pathway. However, in our results the excitatory pathway on average has a shorter latency than the inhibitory pathway. One interpretation is that in addition to the inhibitory pathway being fast, the excitatory pathway is also fast. This would be unexpected because T-stellate cells have a longer latency from sound onset to action potential at a given characteristic frequency compared with GBCs, including in cats (Bourk, 1976; Rhode and Smith, 1986; Young et al., 1988; Blackburn and Sachs, 1989); in gerbils, albeit with a very small difference (Typlt et al., 2012); and in mice (Roos and May, 2012). Another possibility is that MNTB axon branches to MOC neurons are slower-conducting compared with MNTB axon branches to MSO neurons.

Our results pinpoint the segments of the inhibitory pathway responsible for its speed and precision by comparing the relative latency differences, jitter, and response probability between EPSCs and IPSCs evoked using MdL- versus AN-stimulation. Studies have indicated that, although GBCs have large, often suprathreshold PSPs, synaptic summation is required for precise phase-locking and enhanced GBC entrainment in cats (Pfeiffer, 1966; Oertel, 1983; Rhode et al., 1983; Rhode and Smith, 1986; Smith and Rhode, 1987; Joris et al., 1994; Rhode and Greenberg, 1994; Spirou et al., 2005) and mice (Cao and Oertel, 2010) that also involves a low somatic resistance with a short temporal integration window (mice: Oertel, 1983; McGinley and Oertel, 2006; Cao and Oertel, 2010). Here, addition of the CN with AN-stimulation reduces the probability of pathway activity. This could be due to severing of CN projection axons during the slice procedure which could reduce the number of MNTB neurons activated by stimulation. Alternatively, if pathways are intact enough to activate the full complement of MNTB neurons projecting to a given MOC neuron, this lower probability of activity in the inhibitory pathway would confirm that there is not a 1:1 PSP:AP relationship at GBCs, which is more likely in mice compared with cats (Roos and May, 2012), and therefore that synaptic summation contributes to the “coincidence detector” function of GBCs. There may also be species-specific circuit differences, intact inhibitory intrinsic CN circuits, decreased release probability from AN axons, or smaller postsynaptic responses in the GBC. Our observed decreased latency of inhibitory PSCs relative to excitatory PSCs was only apparent when the circuit included the full CN via AN-stimulation, not with MdL-stimulation that lacked GBC participation, indicating that the speed of the inhibitory pathway depends on GBCs. Finally, we consider synaptic jitter as a measure of precision of responses to a stimulus. In our experiments in mice, with MdL-stimulation, the inhibitory pathway has more synaptic jitter than the excitatory pathway. This could be due to GBC→MNTB synaptic jitter, MNTB→MOC synaptic jitter, or combined jitter from the two pathways. However, this is compensated for in the AN-stimulation experiments to result in equal total excitatory and inhibitory jitter. Although in some species T-stellate cells have a small jitter to first AP, perhaps smaller than that of GBCs (cat: Bourk, 1976; Young et al., 1988; gerbil: Typlt et al., 2012), our results suggest that this is not the case in mice; in accordance with the precision of the endbulb of Held→GBC synapse (Kuenzel, 2019), this suggests that this portion of the inhibitory pathway added during AN-stimulation experiments relative to MdL-stimulation experiments is so precise that it can compensate for increased jitter at the next two synapses in the inhibitory pathway.

### Integration of excitation and inhibition

Our findings from both AN-stimulation experiments in wedge-slices and the computational MOC model inform knowledge of the role of integration of synaptic inhibition and excitation on MOC neurons both at the single-cell and population levels. The presence of any inhibition in the model reduced AP rates. In recordings, inhibition was variable and sometimes shorter latency relative to excitation. Then, in the MOC model, changing synaptic timing to reflect the different latencies of EPSCs relative to IPSCs (E–I latency difference) resulted in variable amplitude PSPs and variable AP rates. E–I latency differences that corresponded to values from MOC neuron recordings ranged from having a minimal effect on reducing AP rates when inhibition lags excitation to having the maximal effect when inhibition slightly precedes excitation by ∼1 ms. Interestingly, the most striking example in which inhibition preceded excitation by 4.4 ms did not have the strongest effect on MOC AP rates in the model, suggesting that closely timed synaptic inhibition and excitation are most effective at reducing MOC activity. Further, we speculate that if the timing of inhibition is different across the population of MOC neurons, in addition to the more variable excitatory timing, each cell will have slightly different AP latencies and rates in response to a sound, thus desynchronizing MOC activity across the population.

Our results present a model that while MOC neurons receive rapid and precise excitation at the single-cell level, multiple mechanisms are in place to syncopate, broaden, and smooth activity at the population level. This extends from broadly tuned inhibitory inputs (mouse: Torres Cadenas et al., 2020), variable axonal projection patterns in the cochlea (cat: Liberman and Brown, 1986; guinea pig: Brown, 1987, 1989, 2014, 2016; rat: Vetter et al., 1991; reviewed in Warr, 1992), low synaptic release probability paired with synaptic facilitation at MOC axon terminals onto cochlear OHCs (mouse: Ballestero et al., 2011), and a slow postsynaptic response in the OHC. Our results add fast and precisely timed synaptic inhibition to the list of mechanisms to smooth MOC efferent activity, which paradoxically adds imprecision to the MOC system to exert slow effects on cochlear gain control.
